# Fluid Optimisation in Emergency Laparotomy (FLO-ELA) Trial: study protocol for a multi-centre randomised trial of cardiac output-guided fluid therapy compared to usual care in patients undergoing major emergency gastrointestinal surgery

**DOI:** 10.1186/s13063-023-07275-3

**Published:** 2023-05-06

**Authors:** Mark R. Edwards, Gordon Forbes, Neil Walker, Dion G. Morton, Monty G. Mythen, Dave Murray, Iain Anderson, Borislava Mihaylova, Ann Thomson, Matt Taylor, Marianne Hollyman, Rachel Phillips, Keith Young, Brennan C. Kahan, Rupert M. Pearse, Michael P. W. Grocott, for the FLO-ELA investigators, Alexandra Skubala, Patrick Tapley, Suzanne Kellett, Clare Bolger, Rachel Burnish, Nikki Collings, Andrew Cumpstey, Hannah Wong, Vic Rehnberg, Jessica Lees, Karen Salmon, Naomi Wee, Sarah Harrison, Li Ping Gan, Claire Halloran, Georgios Tsiopanis, Said Seifalian, Richard Webster, Martin Knight, Hannah Theobald, Anna Clark, Thomas Nicholls, James Willey, Sophia Beeby, Luke Bracegirdle, Kate Stoddard, Belinda Roberts, Alice Baker, Norma Diaper, Jonathan Biss, Michael Carter, Francesca Riccio, James Green, Lucy Johnstone, Jade Rand, Kasia Wisniewska, Grant Gibson, Hannah Bateson, Michelle Beveridge, Martyna Marani, Isabel Monger, Agnieszka Burtt, Gary Minto, Iain Christie, Anna Fergusson, Abigail Patrick, Stuart Cleland, Charlotte Eglinton, Natasha Wilmshurst, Fiona Reed, Joanne Smith, Anna Ratcliffe, Elizabeth Freeman, Jennie Kingdon, James Humphreys, Sarah Nelson, Adrian Jennings, Angela Watts, Andrew Moores, Lucy Smith, Jenny Wright, Julian Sonksen, Caroline Moody, Philip Harrington, Jack Lee, Nadim Kozman, Zoe Riddell, Catherine Brennan, Shakira Nathoo, Vikram Anumakonda, Andrea Gait, Richard Pierson, Raj Patel, Lee Plant, Nipun Agarwal, Hadassah Ihlenfeldt, James Heggie, Rachel Olive, Joseph Pick, Sally Hinsley, Nicola Calthorpe, Julie Matthews, Wendy Gardner, Charlotte Topham, Edward Jones, Elliot Yates, Sachin Sekhsaria, Mohamed Amer, Phil Pemberton, Nicholas Coffin, Halden Hutchinson-Bazely, Karen June Pearson, Tracy Edwards, Beth Fitzmaurice, Anna Pierson, Katie Archer, Omar Ahmed, Sajid Khan Mohammed, Alex Hollis, Stephanie Weedon, David Hillier, Joanna Lau, Vishal Amin, Laura Dixon, Joseph Seager, Joe Tyler, Stacey Forsey, N. Parry, Aamer Mughal, Jialuen Goh, Rose Tiller, Daniel Taylor, Hasini Rallage, Alexandra Leech, John Harris, Claire Gabriel, Sheron Clarke, Katherine Pagett, Thomas Rudnick, Nick Brown, Sarah Hare, Eimhear Lusby, Edward Bayliss, Christopher Ward, Rahul Bandopadhyay, Kerrie Wilson, Theodore Floyd, Iram Ahmed, Tom Hatton, Malgorzata Szeszo, Thyra Kyere-Diabour, Daniel Sumner, Tessa Lawrence, Emma Sutton, Winston Ng, Ioannis Kapsokalyuas, Anthony Carter, Anamika Kansal, Leon Bernard, Siew-Ling Harrison, Andrew Feneley, Owen Cooke, Jennifer Hawley, Sophie Berry, Laura Adams, Thomas Hansen, Pieter Bothma, Julie North, Teresa Ferreira, Karan Verma, Karthik Surendran, Aruthy Arumugam, Sunil Jamadarkhana, Carina Cruz, Pearl Baker, Naomi Brice, Antony Ashton, McDonald Mupudzi, Juliette Kemp, Ajay Rahl, Denise Griffin, Aaron Stokes, Keith Ritchie, Arcot Venkatasubramaniam, Robert Cheek, Madonna Brown, Dawn Trodd, Caroline Wrey Brown, Jane Martin, Sam Hammond, Louisa Mason, Nycola Muchenje, Hamish Breach, Amanda Colston, Malcolm Watters, Edwards Miles, Emma Marshall, Madeleine Storey, Victoria Hawley, Edward Gomm, Claire Potter, Melanie Knowles, Edward Beech, Peter van Breda, Helen Langton, Nicholas Suarez, Matthew Rowe, Andrei Tanase, Jonathan Barnes, David Earl, Lorraine Stephenson, Tracy Burdett, Martin Huntley, Emma Cottrell, Hao Ern Tan, Joyce Yeung, Jasraj Kailey, Teresa Melody, Jo Gresty, Julia Sampson, Katie Atterbury, Peter Sutton, Natalie Carling, Eleanor Reeves, Carl Groves, Daniel Crossmann, Sarah Ballinger, Rachel Smith, Marie Thomas, Will Rook, Mohamed Mooradun, Qasim Khan, Arif Qureshi, Llewellyn Fenton-May, Adam Boulton, Daniel Whitney, Shilpa Sannakki, Manekar Avinash, Nikiesha Lee, Neha Sharma, Srinivas Magham, Gareth Moncaster, Rebecca Boulton, Terri-Ann Sewell, Wayne Lovegrove, John Tansley, Nick Watson, Sarah Shelton, Cheryl Heeley, Philip Buckley, Katie Slack, Rebecca Holmes, Andrea Palfreman, Christopher Smith, Mandy Gill, Sue Smith, Tracy Brear, Jill Kirk, Megan Holmes, Camelia Goodwin, Margaret Flynn, Inez Wynter, Kaytie Bennett, Stephen Harris, Corrine Pawley, Patricia Doble, Moira Tait, Richard Gibbs, Tom Edwards, Paul Mackey, Miss Louise Hunt, Jo Hutter, Ed Smyth, Hamish Noble, Thomas Judd, Rose Arkell, Owen Thomas, Karen Watura, Marius Vaida, Suehana Rahman, Saaidullah Sufi, Helder Filipe, Christine Eastgate, Margaret McNeil, Stephen Howey, Glykeria Pakou, Sara Mingo, Amitaa Maharajh, Irina Grecu, Samantha Hammond, Susan Hanson, Julia Ottaway, Victoria Burgess, James Fry, Geoff Watson, Francois Wessels, Hugh Cutler, Arthur Goldsmith, Mark Howes, Subash Sivasubramaniam, Julie Colley, Jenny Porter, K. P. Krishnan, Kerrie Aldridge, Sylvia Willetts, Carol Zullo, Christopher Thompson, Pradeep Shanmugasundaram, Judith Abrams, Katarina Manso, Jamil Razzaque, Sally Scott, Geraldine Hambrook, Elizabeth McKerrow, Tahir Ali, Alastair Ankers, Mohan Ranganathan, Sunil Bellam, Sophie Mason, Paul Marriott, Richard Robley, Bridget Campbell, Penny Parsons, Sean Ramcharan, Susanne Mohamed Anver, Valerie Page, Elaine Walker, Xiaobei Zhao, Laura Osborne, Beena Parker, Rupinder Kaur, Gitana Kulakauskaite-Rasteniene, Mehul Patel, Alastair Lowe, Emma Edmunds, Kieran Hills, Michail Klimovskij, Christopher Ranns, Radha Ganesh, David Jones, Jamie Gibson, Janet Sinclair, Karen Burton, Toni De Freitas, Leon Dryden, Amelia Robinson, Nnamdi Udezue, Tim Faccini, Moon-Moon Majumdar, Kelly Death, Hide Baba, Jaydraman Narendran, Bret Claxton, Andrew Brennan, Louise Akeroyd, Sarah Cooper, Catherine Farrow, Carl Ilyas, James Morgan, Craig Montgomery, Brian Wilkinson, John Dereix, Karen Eaves-Lai, Kate Pye, David Craske, Paul Panesar, Peter Hart, Mark Stubbington, Kim Storton, Kelvin Stewart, Laura Graham, Shereen Bano, Robert Neal, Declan Ryan-Wakeling, Varun Chauhan, Michael Shaw, Maame Aduse-Poku, Soo Jin Kang, Gill Arbane, Kariem El-Boghdadly, Paul Kelly, Jaimin Patel, Marcin Sicinski, Martin John, Mark Ibrahim, Emad Aziz, Sohini Sengupta, Melissa Baldwin, Toby Dixson, Leslie D’souza, Charlotte Taylor, Suneil Rameseur, Heena Bidd, Guy Glover, Andrew Morley, Asta Lukosiute, Anna Janowicz, Tamara Alexander, Joe Lipton, Rathai Anandanadan, Dale Seddon, Alexander Phillips, Louise Davies, Sanjoy Bhattacharyya, Soo Yoon, Sian Fraser, Alex Stilwell, Karin Shoeman, Justin Hews, Sivanth Sivakumar, Floju Chin, Declan Dudley, Gary Colville, Abegail Sawana, Jakob Dudziak, Britta O’Carroll-Kuehn, Chandran Jeganathan, Nicole Richards, Andrew Swain, Charlotte Quamina, Indu Sivanandan, Simon Whiteley, Elizabeth Wilby, Charlotte Quamina, Charlotte Trumper, Kate Varley, Sharmeen Lotia, Eugene Henry, Claire Schofield, Ntima Ntima, Omar Jundi, Amelia Schorah, Luke McMenamin, Robert Jackson, John Jones, Suzie Colquhoun, Joana Faria, Nora Youngs, Aneesha Qadeer, Judith Sharp, Rosie Wragg, Michelle Naylor, Beverley Jackson, Catherine Moriarty, Louise White, Laura Wade, Brian White, Samuel Craven, Coralie Carle, Alan Pope, Mark Chen, Nicola Butterworth-Cowin, Rajneesh Sachdeva, Karen Ellis, Colin Bergin, Emma Reeves, Amy Bamford, Maximina Ventura, Tony Whitehouse, Ronald Carrera, Elaine Spruce, Liesl Despy, Samantha Harkett, Morgan Foster, Antonios Athanasiou, Kamran Malik, Stephanie Porter, Randeep Mullhi, Craig Sadler, James Gasbey, Christopher McGhee, Jignesh Patel, Tracy Mason, Hazel Smith, Alexandra Efimov, Aoife Neal, Stephanie Goundry, Davina Ross-Anderson, Kath MacGloin, James Pennington, Tim Martin, Edyta Niebrzegowska, Mevan Gooneratne, Chhaya Sharma, Neil MacDonald, Jan Whalley, Priyanthi Dias, Gareth Ackland, Peter Shirley, Tim Stephens, Parjam Zolfaghari, Steven Dunkley, Toby Reynolds, Henry Walton, Matthew Smith, Shreya Bali, Sara Hui, Ravi Bhatia, Hew Torrance, Maria Fernandez, Ruzena Uddin, Filipa Santos, Amaia Arrieta, Stephen Barrett, Richard Cashmore, Eleanor Richards, Fatima Seidu, Edward McIlroy, Thomas Urwin, John Samuel, Katherine Brooks, Natalie Gravell, Douglas Blackwood, Tanj Sanghera, Mareena Joseph, Aisha Jalaly, Hannah Nugent, Ben Goodman, Ashley Scott, Ian Clement, Leigh Dunn, Abigail Harrison, Carole Hays, Maite Babio-Galan, Sarah Todd, Lindsay Dawson, Stephanie Henderson, Kapil Arora, Subramani Diwaker, Sam Marcangelo, James Harvey, Mark Dalton, Jeremy Hyams, Tara Shrestha, Kimberley Zwiggelaar, Laura Heggie, Rhona Sinclair, Ben Brown, Sean Cope, Steven Traill, James Durrand, Julie Sheriff, Ashley Allan, Lindsey Woods, Erin Chuter, Rebecca Betts, Rossalyn Thistlethwaite, Elizabeth Turnbull, Monojit Paul, Pete Campbell, Vlad Bashlijski, Charlotte Foley, Amy Ginn, Adam Cookson, Sarah Cornell, Janaki Pearson, Kimberley Rogerson, Ben Eden-Green, Marthin Mostert, Maria Leong, Daniel James Kirkin, Rosie Reece-Anthony, Fatima Ali, Michaela Heller, George Mathew, Beenu Madhavan, Katherine Powell, Noelene Dasey, Waqas Khaliq, Babita Gurung, Cristina Alkhadra, Tarek Mostafa, James Winchester, Megan Thomas, Amit Soodan, Alfa Cresia Nilo, Matthew Bridge, Colette Jones-Criddle, Laura Wilding, Ian Turner-Bone, Ben Morton, Tim Gilbert, Nathan Littley, Natasha Clarke, Greg Moore, Tamryn Miller, Tom Rudnick, Tom Miller, Manab Haldar, Ashok Nair, Michael Jarvis, Precious Basvi, Gillian Bell, Michelle Edwards, Clare Mewies, Stelios Vakis, Emily Burton, Kiran Yelamati, Amit Das, Abhimanyu Bhattacharya, Daniel Massey, Ramkumar Kalaiyarasan, Amro Katary, Win Maung, Dave Robinson, Beth Frost, Samuel Besant, Sunita Gurung, Agah Isguzar, Mina Amirhom, Muhammad Javid, Ashok Raj, Gabrielle Adkins, Rahim Nadeem Ahmed, Josphine Cashman, Elizabeth Smee, Clare Ivermee, Charlotte Cobain, Ana Robles, Yin Choo, Reena Khade, Narayanan Suresh, Lynne Williams, Michele Clark, Pam Race, Anil Agarwal, Rakesh Bhandary, Valasubramaniam Mahadevan, Michael Courtney, James Walker, Susan Hayward, Luke Vamplew, Sally Pitts, Debbie Branney, Andrew Bates, Katie Molloy, Nina Barratt, Sarah Turle, Doug Tunney, Erica Jolly, Tallulah Webb, Katie Bowman, Jessica Kelly, Rebecca Miln, Juan Graterol, Fiona Hammonds, Jessica Summers, Belinda Wroath, Gabbie Young, Benita Adams, Nicki Devooght-Johnson, Eve Fletcher, Michele Wall, Kelly O’Toole, Allwyn Cota, Richard Hunt, Toby Nisbett, Sam Spinney, Tanuja Shah, Brett Doleman, Laura Carrick, Nagendra Prasad, Kathleen Holding, Lianne Hufton, William Speake, Philip Herod, James Nayyar, Daniel Stolady, Shuing Wei, David Daly, Corinne Paxton, Tauseef Ahmed, Anwar ul Huda, Christopher Goddard, Paul Ainsworth, Thomas James Murphy, Barry Jones, Anna Morris, Helen Terrett, John Kirby, Ann Holden, Mark Spiliopoulos, James Hammond, Iain Cummings, Helen Melsom, Louise Duncan, Sameer Somanath, Andrea Kay, Melanie Kent, Michelle Wood, Sarah Clark, Liam O’Hare, Lewis Schofield, Ami Laidlaw, Jordan Minns, James Roe, Stefanie Hobson, Suzanne Naylor, Vicki Atkinson, Phoebe Syme, Lisa Grimmer, Kate Driver, Libby Cole, Denise Webster, John Hickman, Carly Webb, Katie Sweet, Edward Mew, Sarah Warwicker, Susan Tetlow, Alex Middleton, Jonathan Rees, Chris Gough, Sam Howell, Chloe Searles, Shelley Barnes, Louise Seller, Jim Dunham, Alastair Brown, Zoe Garland, Adam Duffen, Thomas Renninson, Anna Chillingworth, Paul Watson, Alex Jones, Rebekah Johnson, Krisztina Kenesey, Thomas Cope, Samuel Fitzpatrick, Annie Amphlett, Christopher Sajoler, James Matthams, Natalie Constable, Jo Poole, Hannah Wilson, Liz Hood, Ruth Greer, James Self, Katherine Nickell, William Headdon, Charlotte Earnshaw, Katie Samuel, Richard Pugh, Jill Andrews, Sarah Evans, Zain Habib, Claudia Variu, Mohammed Zardab, Amy Ellison, Victoria Garvey, Richard Morgan, Shobna Ramakrishnan, Shrisha Shenoy, Michael Kriger, Hefin Llewellyn, Sophie Horrocks, Sam McBride, Rachel Mawley, Alexander Pereiradelima, Tim Cook, Emira Kursumovic, Sarah Hierons, Lucy Howie, Stuart Younie, Lidia Ramos, Tom Cloke, Sara-Catrin Cook, Ben Savage, Alex Dunn, Mark Sheils, Sarah Mitchard, Matthew Gibbins, Olivia Cheetham, Neil Choudhuri, Amelia Davies, Peter Steed, Abigail Harper, Dipayan Choudhuri, Ben Ballisat, Carrie Demetriou, Tim Cominos, Rebecca Powell, Gabrielle Evans, Johannes Retief, Thomas Clark, Jane Montgomery, Rachel Remnant, Ciska Uys, Gabrielle de Selincourt, Sally Ward-Booth, Simon George, Omar Islam, Adam Revill, Daniel Paul, David Portch, Pauline Mercer, Elaine Vandecandelaere, Lorraine Thornton, Victoria Field, Ken Almedilla, Natalie Smith, Jennifer Moran, Thomas Hunt, James Womersley, Raine Thornton, Anne McCarthy, Fleur Rogers, Julie Merizouris, Will Hare, Jonathan Carter, Katie Flower, Greg Warren, Ben Whatley, Virginia Francis, Julie Wollaston, Alex Redome, Louise Cossey, William Spencer, Mike McGovern, Vicky Lewis, Matthew Boyd, Christopher Newell, Sethina Watson, Beverley Faulkner, Emma Gendall, Kati Hayes, Ruth Worner, Elizabeth Goff, Tim Howes, David Cronin, Jacques Carver, Daragh Lehane, Kath Jenkins, Matthew Townsend, Helen Williams, Gemma Nickols, Jodie Garrett, Alexander Jones, Benjamin Savage, Swati Gupta, Mark Dorrance, Edward Lent, Kerry Smith, Dragos Dragnea, Rebecca Williams, Emma Jenkins, Richard Mason, Lydia Osborne, Matt Thomas, Agnieszka Kubisz-Pudelko, Mohamed Gheith, Joanna Allison, Alison Lewis, Kate Beesley, Lucy Pippard, Andrew Shrimpton, Tressy Pitt-Kerby, Jeremy Reid, Nigel Beer, Jess Perry, Matthew Garner, Harriet Noble, Sian Saha, Eleanor Corcoran, John Smith, Evita Pappa, Louise Greig, James Bland, Gudrun Kunst, Elena Stanton, Emma Clarey, Joe Macmillan, Tamsin Rope, John Shenouda, Thomas O’Dell, Hannah Matthews, Temi Adedoyin, Nicola Schunke, Rebecca Mersh, Rosie May, Ashraf Mohammed, Cara Lewis, Eoin Harty, Jonny R. Stephens, Abigail Richardson, Roger Sharpe, Chima Oti, Palitha Bopitiya, James Read, Kiran Chima, Maria Henriksson, Alexa Prichard, Fei Long, John Brandreth, Puvan Suppiah, Winnie Anunda, Kathryn Singh, Claire Ruck, Eleanor Roderick, Isabelle Kamenou, Najwa Soussi, Putul Sarkar, Stefan Wiebe, Yadullah Syed, Aishi Lim, Kerry Barnes, Kwabena Mensah, Aidan Fullbrook, Larry Mulleague, Matt Varier, Viplaw Shukla, Ravi Srinivasan, Najam Pervez, Natasha Schneider, Jasmin Shahnavaz, Duncan Bailey, Rosavic Chicano, Neringa Vilimiene, Shahzaib Ahmad, Neil Richardson, Youssef Mahmoud, Shanni McDonald, Natasha Schumacher, Svetlana Velinova-Teron, Rohit Silhi, Guy Chivers, Julie-Ann Davies, Tracey Cosier, Barry Featherstone, James Rand, Esther Cook, Diana Neely, John Coombes, Harpreet Sodhi, Thomas Burr, Mark Oliver, Michelle Walters, Kim Jemmett, Claudia Dulea, Lucy Cooper, Sam Mcferran, Maxime Rigaudy, Kim Jennett, Heather Weston, Reanne Solly, Emma Ignall, Cathy Praman, Vincent Hamlyn, Gayathri Chinnappa-Srinivas, Angie Organ, Tudor Vlad Moisin, Julia Parnell, Matthew Whitehead, Olivia Hayward, Rosie Malkin, Zoe Bennetton, Anne Devine, Tim Green, Joanna Hubert, Sam Andrews, Deborah Ward, Pauline Brown, Nick Vallotton, Jon Glass, Susan O’Connell, Alice Bevan, Tom Brougham, Lawrie Kidd, Sian Hughes, Jeannine Stone, Deborah Mann, Helen Murray, Fiona Davis, Mark Eveleigh, Jake Hartford-Beynon, Katherine Stratton, Kat Yan Yee Ng, Mandeep Phull, George Joseph, Kalyani Gorrela, Viraj Weerasekera, Nazneen Sudhan, Ayub Khan, Louis Chalmers, Ben Huntley, Sandra Chipperfield, Aparna George, Lace Paulyn Rosario, Tatiana Pogreban, Robert Buhain, Alia Hussain, Asya Veluso Costa, Eleanor Richards, Charles Gibson, Sandeep Kusre, Melanie Hutchings, Sinéad Kelly, Rebecca Pugsley, Hamza Malik, Alec Beaney, Tom Woodward, Zahra Essackjee, Kangni Chen, Bruce McCormick, Cath Matthews, Michelle Walter, Elizabeth Gordon, Sadie Heddon, Timothy Warrener, Peter Valentine, Joel Prescott, Samantha Keenan, Emily Johnson, Eleanor Higgs, Jessica Thrush, Laura Tulloch, Fiona Osborne, Victoria Poyntz, Pooja Takhar, Connie Rowlands, Michael Mcalindon, Victoria Lacey, Nicholas Cowley, Stephanie Chamberlain, Sally Rudge, Sian Bhardwaj, David Freeman, Brendan Spooner, Satinder Dalay, Nick Fitton, Rebecca Davies, Kay Fisher, Laura Naumann, Elma Wong, Simon Leach, Helen Moore, Rebecca Wilcox, Rhys Parry, Alison Magness, Mandy Carnahan, Matthew Travis, Colene Adams, Samuel Passey, Richard Colebrook, David Elcock, Priscilla Mhembere, Jayne Rankin, Yee Yin Cheng, Chris Clulow, Jo Stickley, Anne Carter, Alison Stephens, Elizabeth Buckingham, Laura Price, Ryan Jones, Andy Taylor, Maria Ochoa-Ferraro, Harriette Beard, Jeremy Corfe, Jocelyn Keshet-Price, Deidre Fottrell-Gould, Gill Foot, Lisa Hudig, Karen Convery, Martyn Oliver, Hannah Neil, Georgina Randell, Melanie Maxwell, Kavitha Kuntumalla, Pushpaj Gajendragadkar, James Wu, Danielle Huckle, Stephen Petley, Nadine Jones, Karen Rahilly, Gail Williams, Margaret Coakley, Laura Jones, Dominic Manetta-Jones, Sara Churchill, Laura Fulton, Suyogi Jigajinni, Emma Collins, Fillipa Santos, Noah John, Abhilash Das, Christopher Manville, Tom Abbott, Hester Carter, Lina Kanapeckaite, Gavin Stead, Jonathan Holmes, Amy Ireson, Edward Gill, Stephanie Kwok, Alastair Duncan, Hannah Greenlee, Ian Venables, Rose Jama, Iain Moppett, Cecilia Peters, Lucy Ryan, Louise Conner, Megan Meredith, Amy Clark, Abi Noah, Louise Potter, Will Lindsay, Jaina Parmar, Dan Harvey, David Evans, Marc Chikhani, Ben Lowe, Kevin O’Donoghue, Avninder Chana, Rachel Roke, Ioannis Tsagurnis, Harvey Dymond, David Sleep, Kristina Owens, Rachel Beer, Dawn Simmons, Donna Cotterill, Ime Eka, Sandra Beech, Pei Jean Ong, David Ritchie, Susan Wilkinson, Matthew Butler, David Crossley, Victoria Van Der Schyff, Irum Ghazanfar, Andrew Mawer, Ana Almeida, Lucy Duggal, Jonathan Lightfoot, Anna Simpson, Susan O’Connell, Tom Rennison, Robert Thompson, Susan Fowler, Sandra Pirie, Patricia Cochrane, David Nesvadba, Patrice Forget, Pauline Ganley, Jennifer Noble, Amanda Coutts, Sue Jackson, Tim Prescott, William Smith, Anne Harrison, Emily Omuwie, Rachel Johnson, Jennifer Evans, Rebecca Rudd, Isobel Loeffler, Catarina Veiga, Poh Choo Teoh, Samuel Chambers, Oleg Bumbac, Ross Holcombe-Law, Alexandria Page, Paul Jackson, Daniel George, Tess Wilkinson, Daniel Kirkin, Michaela Lloyd, Leanne Smith, Amie Reddy, William McCaig, Harriet Murrant, Ammara Masoud, Mia Davis, Debasis Pradhan, Joanne Rudkin, Matthew Byrne, Georgios Tsinaslaniois, Vasileios Bafitis, Christopher Black, Cassandra George, Marketa Keltos, Maria Letts, Victoria Allinson, Angela Foulds, Sophie Gittus, Lakshmi Aneesh, Ayman Nash, Prathiban Kumar, James Graham, Andrew Donnaly, Martin Grigg, Ariana Singh, Aastha Chawla, Calum McGrady, Nicola Walker, Christopher Brennan

**Affiliations:** 1 Department of Anaesthesia, Southampton General Hospital, University Hospital Southampton NHS Foundation Trust, Tremona Road, Southampton, SO16 6YD UK; 2 Perioperative & Critical Care Research Group, NIHR Southampton Biomedical Research Centre, University Hospital Southampton NHS Foundation Trust / University of Southampton, Southampton, UK; 3 Department of Biostatistics & Health Informatics, King’s College London, London, UK; 4 Pragmatic Clinical Trials Unit, Queen Mary University of London, London, UK; 5 Academic Department of Surgery, University of Birmingham, Birmingham, UK; 6 University College London Hospitals NIHR Biomedical Research Centre, London, UK; 7 James Cook University Hospital, Middlesbrough, UK; 8 Salford Royal NHS Foundation Trust, Salford, UK; 9 University of Manchester, Manchester, UK; 10 Health Economics and Policy Research Unit, Wolfson Institute of Population Health, Mary University of London, London, Queen UK; 11 Nuffield Department of Population Health, University of Oxford, Oxford, UK; 12 Department of Critical Care, University Hospitals Dorset NHS Foundation Trust, Poole, UK; 13 Department of Surgery, Musgrove Park Hospital, Taunton, UK; 14 School of Public Health, Imperial College London, London, UK; 15 London, UK; 16 MRC Clinical Trials Unit at UCL, University College London, London, UK; 17 Faculty of Medicine & Dentistry, Mary University of London, London, Queen UK

**Keywords:** Emergency surgical procedures/adverse effects, Hemodynamics/physiology, Intraoperative/methods, Postoperative complications/prevention and control, Prospective studies

## Abstract

**Introduction:**

Postoperative morbidity and mortality in patients undergoing major emergency gastrointestinal surgery are a major burden on healthcare systems. Optimal management of perioperative intravenous fluids may reduce mortality rates and improve outcomes from surgery. Previous small trials of cardiac-output guided haemodynamic therapy algorithms in patients undergoing gastrointestinal surgery have suggested this intervention results in reduced complications and a modest reduction in mortality. However, this existing evidence is based mainly on elective (planned) surgery, with little evaluation in the emergency setting. There are fundamental clinical and pathophysiological differences between the planned and emergency surgical setting which may influence the effects of this intervention. A large definitive trial in emergency surgery is needed to confirm or refute the potential benefits observed in elective surgery and to inform widespread clinical practice.

**Methods:**

The FLO-ELA trial is a multi-centre, parallel-group, open, randomised controlled trial. 3138 patients aged 50 and over undergoing major emergency gastrointestinal surgery will be randomly allocated in a 1:1 ratio using minimisation to minimally invasive cardiac output monitoring to guide protocolised administration of intra-venous fluid, or usual care without cardiac output monitoring. The trial intervention will be carried out during surgery and for up to 6 h postoperatively. The trial is funded through an efficient design call by the National Institute for Health and Care Research Health Technology Assessment (NIHR HTA) programme and uses existing routinely collected datasets for the majority of data collection. The primary outcome is the number of days alive and out of hospital within 90 days of randomisation. Participants and those delivering the intervention will not be blinded to treatment allocation. Participant recruitment started in September 2017 with a 1-year internal pilot phase and is ongoing at the time of publication.

**Discussion:**

This will be the largest contemporary randomised trial examining the effectiveness of perioperative cardiac output-guided haemodynamic therapy in patients undergoing major emergency gastrointestinal surgery. The multi-centre design and broad inclusion criteria support the external validity of the trial. Although the clinical teams delivering the trial interventions will not be blinded, significant trial outcome measures are objective and not subject to detection bias.

**Trial registration:**

ISRCTN 14729158. Registered on 02 May 2017.

**Supplementary Information:**

The online version contains supplementary material available at 10.1186/s13063-023-07275-3.

## Administrative information

**Table:** 

**Data category**	**Information**
Primary registry and trial identifying number	ISRCTN registry ISRCTN 14729158
Date of registration in primary registry	02 May 2017
Secondary identifying numbers	IRAS 214459, HTA 15/80/54, CRI0336
Source(s) of monetary or material support	NIHR Health Technology Assessment Programme, HTA (HTA 15/80/54)
Primary sponsor	University Hospital Southampton NHS Foundation Trust, UK
Secondary sponsor(s)	N/A
Contact for public queries	Ms Aga Burtt, admin@floela.org
Contact for scientific queries	ME, mark.edwards2@uhs.nhs.uk
Public title	A clinical trial of blood flow optimisation for patients who have emergency bowel surgery
Scientific title	*FLuid Optimisation in Emergency LAparotomy (FLO-ELA): an open, multi-centre, randomised controlled trial of cardiac output-guided haemodynamic therapy compared to usual care in patients undergoing emergency bowel surgery*
Countries of recruitment	United Kingdom
Health condition(s) or problem(s) studied	Emergency gastrointestinal surgery
Intervention(s)	Intervention: minimally invasive cardiac output monitoring to guide protocolised administration of intravenous fluid during and for up to six hours after major emergency bowel surgery
	Control: standard care without the use of cardiac output monitoring
Key inclusion and exclusion criteria	Ages eligible for study: ≥ 50 years Sexes eligible for study: both Accepts healthy volunteers: no
	Inclusion criteria: Patients aged 50 years and over undergoing an expedited, urgent or emergency major abdominal procedure on the gastrointestinal tract eligible for inclusion within the National Emergency Laparotomy Audit (NELA)
	Exclusion criteria: Refusal of patient consent, clinician refusal, abdominal procedure outside the scope of NELA, previous enrolment in the FLO-ELA trial, previous inclusion in the NELA audit within the same hospital admission, current participation in another clinical trial of a treatment with a similar biological mechanism
Study type	Interventional
	Allocation: randomised intervention model. Parallel assignment masking: open label
	Primary purpose: prevention
	Phase III
Date of first enrolment	September 2017
Target sample size	3138
Recruitment status	Recruiting
Primary outcome(s)	Number of days alive and out of hospital within 90 days of randomisation
Key secondary outcomes	Mortality within 90-days and 1 year of randomisation, duration of postoperative hospital stay, duration of postoperative critical care unit stay, hospital readmission within 90-days of randomisation

## Background

Emergency abdominal surgery on the gastrointestinal tract (laparotomy) is a common major surgical procedure performed for life-threatening abdominal conditions such as bowel obstruction or bleeding due to underlying cancer, infection, or previous surgery. It is performed on over 30,000 patients in England and Wales each year [1, 2] and has a particularly high burden of postoperative morbidity and mortality, with a 90-day postoperative mortality rate of 18–20% in those aged 50 and over [2, 3]. The critical need to improve the care of patients undergoing this procedure has been recognised in the establishment of the National Emergency Laparotomy Audit (NELA), a national audit of care and outcomes in this patient group [1, 2], and a number of national quality improvement initiatives [4, 5].

Intra-venous fluids given during and after surgery have an important effect on patient outcomes, in particular following major gastrointestinal surgery [6]. They are commonly prescribed in relation to subjective criteria leading to wide variation in clinical practice [7]. The use of cardiac output monitoring to guide intra-venous fluid dosing as part of a haemodynamic therapy algorithm has been studied for many years and has been shown to modify inflammatory pathways and to improve tissue perfusion and oxygenation [8, 9]. An updated meta-analysis of this intervention in predominantly elective surgery incorporated the largest contemporary trial in this area [10]. Complications were less frequent among patients treated according to a hemodynamic therapy algorithm (intervention 488/1548 [31.5%] vs controls 614/1476 [41.6%]; risk ratio (RR) 0.77 [0.71–0.83]). Duration of hospital stay was reduced (mean reduction 0.79 days [0.62–0.96]). There was a non-significant reduction in mortality at the longest follow-up (intervention 267/3215 deaths [8.3%] vs controls 327/3160 deaths [10.3%]; RR 0.86 [0.74–1.00]; *p* = 0.06).

These findings are not directly generalisable to patients undergoing emergency abdominal surgery due to fundamental pathophysiological differences. In the emergency setting, there may be acute inflammation, sepsis, bleeding, and fluid disturbances established before surgery begins. There are similarities with critically ill patients, in whom the benefit of fluid resuscitation based on cardiac output monitoring is uncertain [11–13]. There is a lack of adequately powered, multicentre studies of this treatment in emergency surgical patients.

The aim of this trial is to evaluate the effects of perioperative haemodynamic therapy guided by cardiac output on the number of days spent alive and out of hospital following major emergency bowel surgery. NELA, commissioned by the Healthcare Quality Improvement Partnership (HQIP) as part of the National Clinical Audit Programme, provides a detailed ongoing dataset and engaged clinical community in this patient group. This supports an efficient trial design with minimal supplementary data collection beyond that already collected routinely for NELA and national databases. Composite outcomes of mortality and time spent in hospital are efficient, patient-centred postoperative outcome measures recommended in perioperative core outcome sets [14, 15]. Days alive and out of hospital within 90 days of randomisation was selected as an outcome measure that is of clear importance to patients and healthcare systems, is expected to be modifiable by this intervention, and is statistically efficient.

### Objectives

We hypothesise that in patients aged 50 and over undergoing major emergency gastrointestinal surgery, cardiac output-guided fluid therapy will increase the number of days spent alive and out of hospital within 90 days of randomisation when compared with usual care. Secondary hypotheses are that this intervention will reduce all-cause mortality within 90 days and 1 year. We will evaluate whether the intervention is cost-effective. A 12-month internal pilot tested the feasibility of site and participant recruitment, representativeness of participants recruited, and protocol compliance (see Additional file 1).

## Methods

The study protocol is reported in line with SPIRIT guidelines (see Additional file 2: SPIRIT checklist) [16].

### Study design

Multi-centre, open, two-arm, parallel-group randomised controlled trial with an internal pilot.

### Setting

Emergency surgical services of up to 50 hospitals in the UK. Participant recruitment started in September 2017. Recruiting site eligibility criteria include having surgical services performing major emergency gastrointestinal surgery in adults, participation in the National Emergency Laparotomy Audit (NELA – sites in England and Wales only), the ability to provide cardiac output monitored haemodynamic therapy, and previous participation in interventional research. See Additional file 1 for a list of active sites.

### Participants

#### Inclusion criteria

Patients aged 50 years and over, with an NHS/Community Health Index (CHI)/Health and Care (H&C) number, scheduled to undergo a surgical procedure which fulfils the criteria for entry into NELA, i.e. an expedited, urgent or emergency abdominal procedure on the gastrointestinal tract within the audit scope. For a full list of procedures within the audit scope, see Additional file 1.

#### Exclusion criteria

Refusal of patient consent, clinician refusal, previous enrolment in the FLO-ELA trial, previous inclusion in NELA within the current hospital admission, current participation in another clinical trial of a treatment with a similar biological mechanism, scheduled abdominal procedure outside the scope of NELA (see Additional file 1).

### Enrolment and randomisation

Strategies for achieving adequate participant enrolment include ensuring the target number of recruiting sites is achieved, co-ordinated multi-disciplinary trial leadership at a regional and hospital level, and selecting sites with experienced local investigators and research teams. National trainee research networks have been engaged to help identify and recruit patients presenting outside normal working hours [17].

Potential participants will be screened by staff at the site having been identified from operating theatre lists and by communication with clinical teams. Consent to trial participation is sought, and most eligible patients will have the capacity to consent [18, 19]. An authorised member of the team will be responsible for obtaining written informed consent. This process will include provision of a patient information sheet accompanied by the relevant consent form (both documents available at www.floela.org/Study-Documents), and an explanation of the aims, methods, anticipated benefits, and potential harms of the trial. Consent will cover necessary data collection and linkage.

The trial will also include participants who are incapable of giving consent due to severe pain, opioid analgesics, multiple medical interventions, the requirement for surgery within a short timeframe, prioritisation of medical information, or lack of mental capacity due to delirium or sedation. These patients may be entered into the trial via consultation and agreement from a Personal or Nominated Consultee (England, Wales, and Northern Ireland) or by gaining consent from a guardian or welfare attorney (Scotland). In England, Wales, or Northern Ireland, if consultation is not possible, due to the emergency nature of this treatment, the patient may be enrolled via emergency consent, based on consultation and agreement from an independent doctor nominated by the local research team. In all cases where the patient has not provided informed consent themselves prior to trial enrolment and regains capacity after surgery, retrospective consent will be sought. See Additional file 1 for full details. Eligible patients who are not entered into this trial will be recorded in local screening logs.

#### Randomisation

After enrolment but before the start of surgery, participants will be allocated to treatment groups in a 1:1 ratio using a computer-generated minimisation algorithm with a random component. The minimisation factors will be patient age (50–64 years, 65–79 years, and 80 + years) and ASA class (I, II, III, IV, and V). Randomisation will be performed by research staff as close as possible to the start of anaesthesia, typically when the patient arrives in the theatre suite for surgery. Randomisation is provided by a secure central online service.

### Study interventions

The trial treatment period will commence at the start of general anaesthesia and continue for up to 6 h after the completion of surgery. Eligible patients will be randomised to receive either cardiac-output guided haemodynamic therapy (intervention group), or usual care without cardiac output monitoring. Perioperative management for *all* patients during the trial treatment period will be in accordance with recommended guidance below.

#### Perioperative management for all patients

Care for all patients has been loosely defined to avoid extremes of clinical practice but also practice misalignment [20]. All patients will receive standard measures to maintain oxygenation (SpO_2_ ≥ 94%), haemoglobin (> 80 g/L), and core temperature (36.5–37.5 °C). A list of recommended fluids that may be given will be provided (see Additional file 1). These fluids have a composition recommended by NICE for their specific clinical indication, i.e. maintenance fluid requirements or plasma volume expansion [21]. A recommended “maintenance” fluid will be administered at 1 ml/kg/h. Mean arterial pressure will be maintained between 60 and 100 mmHg using a vasopressor or vasodilator as required. If inotropes, vasoconstrictors, or vasodilators are required, they should be provided by intravenous infusion rather than intermittent bolus. Other aspects of perioperative care should be based on the best available evidence for this group [22, 23], and the audit standards recommended by NELA [2].

#### Control group

Patients in the control group will be managed by clinical staff according to usual practice, without the use of cardiac output monitoring. In addition to the maintenance fluid, 250-ml fluid challenges with a recommended intra-venous fluid will be given for plasma volume expansion (see Additional file 1). These will be administered at the discretion of the clinician guided by pulse rate, arterial pressure, urine output, core-peripheral temperature gradient, serum lactate, and base excess. Patients should not be randomised if the clinician intends to use cardiac output monitoring regardless of study group allocation; this is considered “clinician refusal” and is a specific exclusion criterion. However, clinical staff are able to request cardiac output monitoring if this is required to inform the treatment of a patient who becomes critically ill (e.g. because of severe haemorrhage); in this situation, a protocol deviation form will be completed.

#### Intervention group

**Fig. 1:**
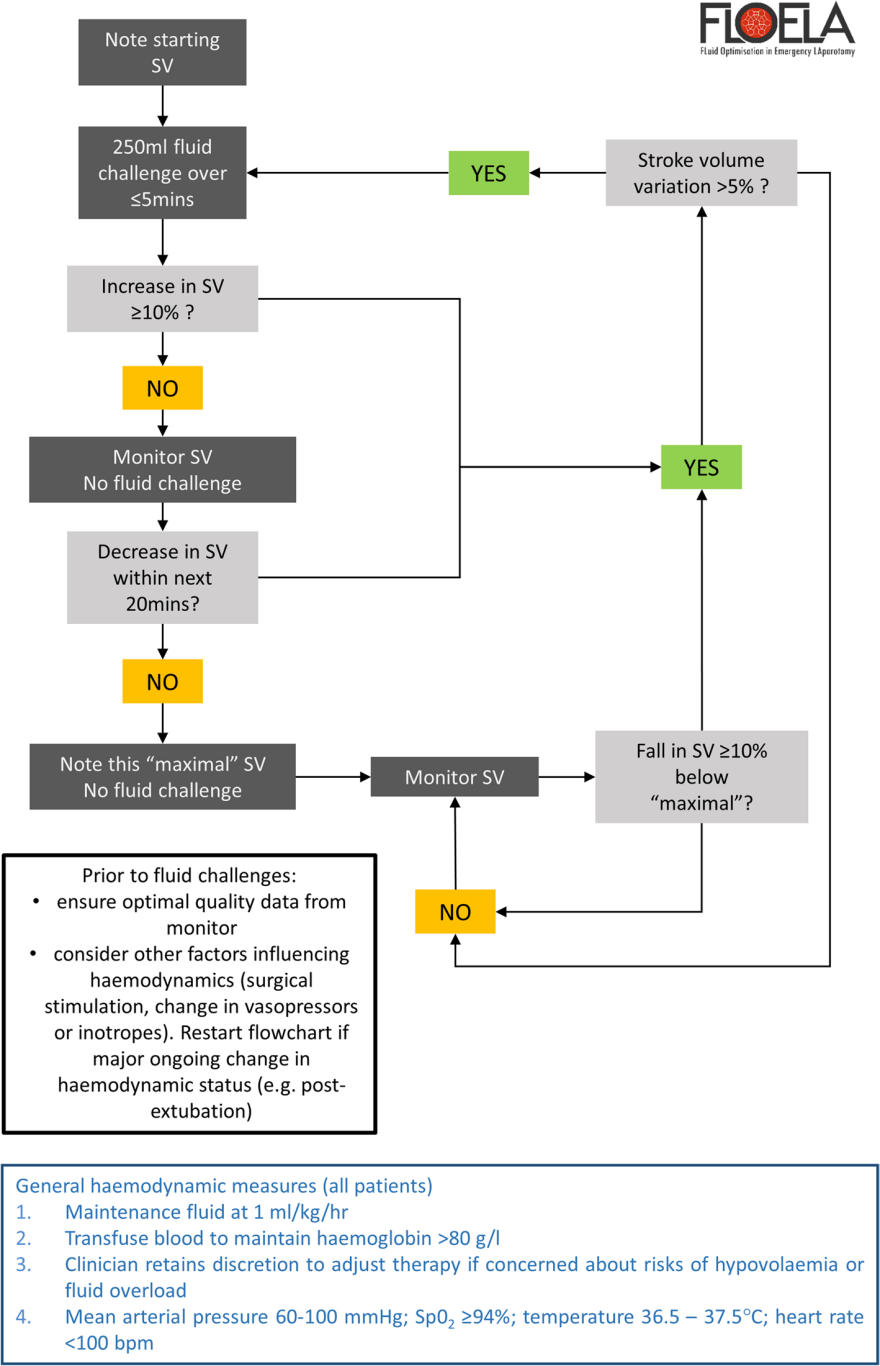
Algorithm for cardiac output-guided haemodynamic therapy for participants in the FLO-ELA intervention group

The trial intervention and haemodynamic algorithm was developed by the FLO-ELA trial group. It is based on best available contemporary evidence in this area to inform aspects such as device and fluid choices, duration of the intervention, and haemodynamic targets [10, 21, 24, 25]. The cardiac output-guided haemodynamic therapy intervention will commence with the induction of anaesthesia and continue at least until the end of surgery. In patients receiving level 2/3 critical care after surgery, the intervention will continue for 6 h after the end of surgery. This level of care may be delivered in intensive care units, high-dependency units, or post-anaesthetic care units (PACU). For patients with a clinical plan to be transferred to level 1 (ward) care after initial recovery from anaesthesia in the PACU, wherever possible the intervention should be delivered for 6 h within the PACU before transfer. See Additional file 1 for definitions of levels of care. Cardiac output and stroke volume will be measured by a cardiac output monitor. Clinicians may choose from a range of cardiac output monitors in established use which have been shown to track changes in cardiac stroke volume accurately. See Additional file 1 for a recommended list. No more than 500 ml of intra-venous fluid will be administered within the intervention period prior to commencing cardiac output monitoring. In addition to the maintenance fluid, patients will receive a 250-ml fluid challenge with a recommended intra-venous fluid administered over 5 min or less. This fluid challenge will be repeated if there is evidence of fluid responsiveness, defined as ≥ 10% increase in stroke volume in response to the previous fluid challenge AND stroke volume variation (SVV) > 5%. This will continue until a maximal value of stroke volume is achieved, defined as a stroke volume maintained for at least 20 min with no evidence of fluid responsiveness. See Fig. 1. Following major changes in haemodynamic status, such as following emergence from anaesthesia, further 250-ml fluid challenge is recommended to re-establish the presence or absence of fluid responsiveness, and the maximal value of stroke volume revised if necessary. All other management decisions will be taken by clinical staff. If there is a clear clinical indication, the treating clinician may adjust both the volume and type of fluid administered, for example if there is concern about persistent hypovolaemia or fluid overload based on clinical circumstances or physiological measurements.

### Blinding and procedures to minimise bias

FLO-ELA is a pragmatic trial of a treatment algorithm. It is not possible to conceal treatment allocation from all staff in trials of this type. Therefore, this trial will be open-label, and patients and the staff delivering the intervention will be unblinded. However, procedures will be put in place to minimise bias. Clinicians will be instructed that the decision to admit a patient to critical care after surgery should be made on conventional clinical grounds before randomisation. Confirmation of the primary and secondary outcomes is objective and automated through the use of routine national health databases. While hospital discharge date may be influenced by potentially unblinded clinicians, the risk of bias is low as separate teams are involved in delivering the intervention (anaesthesia/critical care) and overseeing later postoperative recovery and discharge (surgeons). The latter will typically be unaware of group allocation and discharge decisions are made on average 10–14 days after the trial intervention has been completed. Adjudication of serious adverse events (SAEs) will be by the local Principal Investigator, who will be blinded to study group allocation.

Research staff enrolling patients will not necessarily be blinded to previous allocations but the randomisation method used is not predictable so there is little risk of selection bias [26]. The trial management group and the trial steering committee will not see results broken down by treatment arm during the trial. The trial statisticians and health economists will not have access to unblinded trial data (i.e. data with treatment allocation included, or variables which could predict treatment allocation such as compliance) until after the final statistical analysis plan and health economics analysis plan have been signed off and the database is locked for final analysis. The independent data monitoring committee will see the outcome results by treatment group but the report will be prepared by an independent statistician.

### Data collection

At hospitals in England and Wales, data are collected for NELA on a secure online web portal as part of routine care, under Sect. 251 of the NHS Act 2006. Data completeness is fed back to sites regularly as an audit standard. A small number of data fields will be added to the NELA web portal for FLO-ELA trial participants. Identifiable data held in the trial randomisation system will be linked to national databases to obtain outcome data. Outcomes data will be merged with pseudonymised NELA data for statistical and health economic analyses. Data sharing agreements will be established with HQIP, NHS Digital, and devolved nation equivalents and are described in consent materials. In Scotland and Northern Ireland, an electronic CRF database will be produced with identical data fields to those used in NELA/FLO-ELA.

### Trial outcomes

#### Primary end point

Number of days spent alive and out of hospital within 90 days of randomisation (DAOH-90).

#### Secondary end points

Mortality within 90 days and 1 year of randomisation.

#### Process measures

1. Duration of hospital stay (number of days from randomisation until hospital discharge)
2. Duration of stay in a level 2 or level 3 critical care bed within the primary hospital admission post-randomisation
3. Hospital readmission as an inpatient (overnight stay) within 90 days from randomisation

#### Health economic outcomes

1. Mean cost of index hospital admission (including haemodynamic therapy) in intervention and control-allocated participants
2. Mean cost of secondary care within 90 days from randomisation
3. Mean cost of secondary care within one year from randomisation
4. Quality-adjusted life years (QALYs) at 90 days from randomisation using EQ-5D-3L utility estimated from the EPOCH trial participant data [4] and FLO-ELA participants’mortality data
5. Quality-adjusted life years (QALYs) at 1 year from randomisation using EQ-5D-3L utility estimated from the EPOCH trial participant data [4] and FLO-ELA participants’mortality data
6. Cost-effectiveness of cardiac output-guided fluid therapy at 90 days from randomisation
7. Cost-effectiveness of cardiac output-guided fluid therapy at 1 year from randomisation

#### Internal pilot outcomes

1. Number of active recruiting sites.
2. Number of participants randomised.
3. Protocol compliance (intervention group adherence and control group contamination).
4. Representativeness of the participants recruited compared with all eligible NELA patients with respect to age, sex, and preoperative physiological markers.

### Assessment of outcomes

DAOH is a validated postoperative outcome measure calculated as a composite of postoperative mortality, length of index hospital stay post-randomisation, and the duration of any hospital readmissions. DAOH-90 will be calculated as follows [27]:

- Participants who die within the 90 days following randomisation will be allocated a value of zero days
- For participants surviving to 90 days: DAOH-90 = 90 − (number of days spent in hospital within 90 days of randomisation)

The number of days in hospital is defined as an inpatient (overnight) stay in any hospital. It is made up of the initial postoperative stay in hospital for surgery (the number of days from randomisation until the patient is discharged) as well as any hospital readmissions (number of days spent in any hospital after discharge) up to day 90 after randomisation.

We will request hospital episode statistics and mortality data from NHS Digital for participants in England or equivalents for the devolved nations using identifiable data collected in the trial randomisation system (see Additional file 1). Duration of postoperative hospital and critical care stay (during the index hospital admission) will be derived from NELA data.

### Baseline and other follow-up data

Data on baseline demographic and clinical participant characteristics, perioperative events, and details of the trial intervention will be collected by a review of the participant’s medical records (see Additional file 1).

The schedule of enrolment, interventions, and assessments is summarised in Table 1.

**Table 1:**
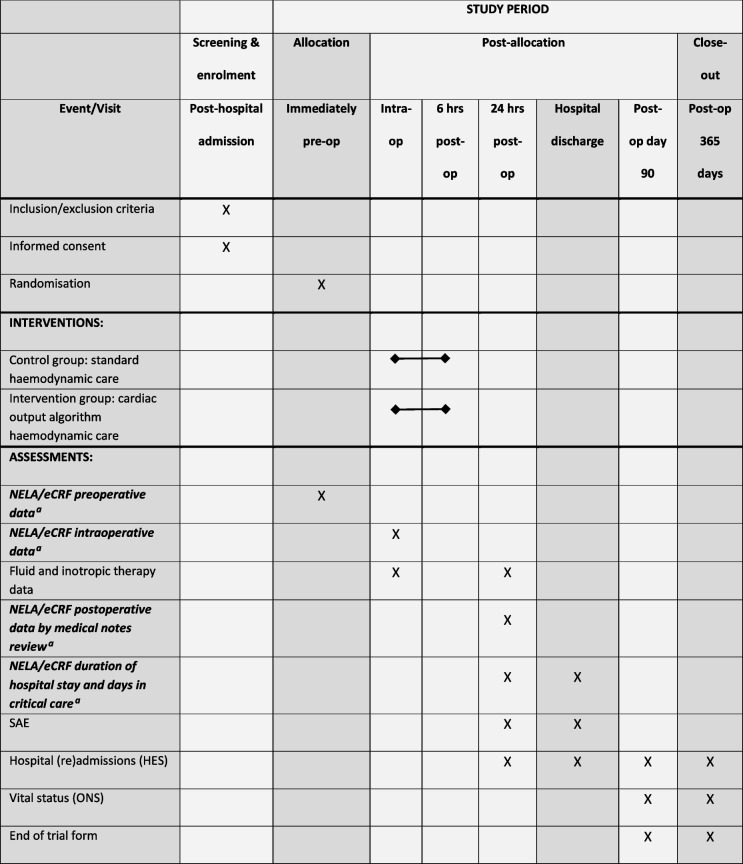
Schedule of enrolment, interventions, and assessments for participants in the FLO-ELA trial

^a^These data are already collected as routine care by medical teams for NELA

### Protocol compliance monitoring

Predefined protocol deviations that will be reported include failure to use cardiac output monitoring in an intervention group patient, failure to follow the haemodynamic algorithm (defined as at least one cycle of fluid bolus with measurement of stroke volume response) in an intervention group patient when a cardiac output monitor is being used, and the use of cardiac output monitoring in a control group patient, including forms of monitoring based on stroke volume variation or pulse pressure variation only. Protocol deviations will be monitored and feedback given to centres with high levels of non-compliance.

### Estimands

An estimand is a precise definition of the treatment effect to be estimated for a given outcome. It describes the treatment effect’s interpretation using a standard framework (see Table 2) in order to increase clarity around the interpretation of study results [28, 29]. The estimand for the primary outcome, DAOH-90, is shown in Table 2. Briefly, the estimand is the ratio of means of DAOH-90 between protocolised cardiac output-guided haemodynamic therapy vs. usual care, regardless of adherence or use of cardiac monitoring in the control arm, in participants aged ≥ 50 years who would undergo emergency bowel surgery under assignment to either treatment. Estimands for the secondary outcomes (mortality at 90 or 365 days) are the same as the estimand for the primary outcome, except for the different endpoints, and an odds ratio will be used as the summary measure.

**Table 2:** Estimand for the primary outcome (DAOH-90). “Intercurrent event” denotes a post-randomisation event which may affect the interpretation or occurrence of outcome data (e.g. failure to receive treatment as intended, using a different treatment to the one assigned, etc.). Principal stratum and treatment policy are strategies to handle intercurrent events in the estimand definition. Here, the principal stratum strategy denotes that interest lies in the subpopulation of patients who would undergo emergency surgery regardless of their treatment allocation, and the treatment policy strategy denotes that the intercurrent event (e.g. failure to initiate cardiac output monitoring, the intervention algorithm not being followed) is considered part of the treatment strategy and interest lies in evaluating the treatment regardless of the occurrence of such events

**Aspect**	**Definition**
**Target population**	Patients ≥ 50 years old who undergo emergency bowel surgery
**Endpoint**	Days alive and out of hospital within 90 days of randomisation (DAOH-90 = count of days alive and out of hospital within 90 days of randomisation where DAOH-90 = 0 if patient dies within 90 days and DAOH-90 = 90 − (days in hospital within 90 days of randomisation) if patient alive 90 days after randomisation)
**Treatment conditions**	**Intervention group—**Protocolised cardiac output-guided haemodynamic therapy during surgery, and for 6 h after, regardless of whether the haemodynamic protocol has been followed correctly **Usual care group—**Intravenous fluid administration without the use of cardiac output monitoring or protocol
**Summary measure**	Ratio of means (Intervention vs. usual care group)
**Intercurrent events**	**Strategy**
Surgery not received (applies to both treatment arms)	Principal stratum (of participants who would undergo surgery regardless of treatment allocation)
Procedure modified after surgery begins such that no longer eligible for NELA (applies to both treatment arms)	Treatment policy
Receipt of cardiac output monitoring (control arm only)	Treatment policy
Failure to initiate cardiac output monitoring during/after surgery (intervention arm only)	Treatment policy
Cardiac output monitoring initiated but intervention algorithm not followed	Treatment policy

### Sample size

3138 participants (1569 per group) will be required to detect a 3.2-day increase in DAOH-90 (from mean 64.5 (SD 28.0) days in the control group to 67.7 (SD 27.1) days in the intervention group), with 90% power, a 5% alpha level, and a 2% dropout rate. The parameter choices for this sample size calculation are included in Additional file 1.

### Statistical analysis

#### Summary of baseline data and participant disposition

The number of patients recruited and followed up will be recorded in a CONSORT flow chart. Baseline characteristics will be summarised by treatment group.

#### General analysis principles

The analysis strategy has been chosen to facilitate the estimation of the estimands described above. Participants will be analysed according to the treatment group to which they were randomised. All eligible participants for whom an outcome is available will be included in the analysis [30], with the following exceptions. First, participants who were randomised in error (i.e. were ineligible at the time of randomisation) will be excluded from the analysis. This is because those participants fall outside the target population, so their inclusion could bias results away from the target treatment effect. We note that because eligibility is assessed before randomisation, excluding these participants poses no threat to internal validity. Second, participants who were eligible and randomised but did not ultimately undergo surgery will also be excluded from the analysis. This is to estimate the principal stratum strategy described in Table 2 (i.e. to estimate the treatment effect in the subset of participants who would undergo surgery under either treatment allocation) [31]. The approach of excluding these participants from the analysis will be unbiased under the assumption that a participant’s treatment allocation will not affect whether they ultimately undergo surgery (i.e. that participants whose surgery is cancelled in the usual care group would also have had their surgery cancelled if assigned to the intervention, and vice versa). This assumption is justified on the basis that (i) given the serious nature of the condition, it is implausible that surgery would be cancelled based on the allocated method of fluid delivery, and (ii) the surgeons involved in deciding whether the procedure should go ahead will typically be unaware of the specific method of fluid delivery to be used during surgery.

For each analysis, we will present the number of patients included in the analysis, a summary measure of the outcome in each treatment group, treatment effect, 95% confidence interval, and a two-side *p*-value. *P*-values < 0.05 will be considered statistically significant.

#### Primary outcome analysis

The primary outcome (days alive and out of hospital within 90 days of randomisation) will be analysed using a mixed-effects negative binomial regression model, with a random-intercept for centre [32]. The model will be adjusted for the minimisation factors of patient age and ASA class (I, II, III, IV, and V) [33], as well as the following prognostic baseline covariates: urgency of surgery (immediate, urgent, and expedited), Glasgow Coma Score (GCS), systolic blood pressure, and pulse rate [34]. Urgency of surgery and ASA class will be included as categorical variables, while patient age, GCS, systolic blood pressure, and pulse rate will be included as continuous variables. Patient age and GCS will be included assuming a linear association with the outcome, and systolic blood pressure and pulse rate will be included using restricted cubic splines with 3 knots (knots will be placed based on Harrell’s recommended percentiles) [35, 36]. Missing baseline data will be handled using mean imputation for continuous variables, and a missing indicator variable for categorical variables [37].

#### Secondary outcome analysis

Mortality within 90 days and 1 year of randomisation will be analysed using an analogous mixed-effects logistic regression model (same random effects and covariate strategy as primary outcome). Duration of hospital stay and hospital re-admission will be analysed using a competing-risk time-to-event model, which includes mortality as a competing risk [38]. Both models will adjust for the set of covariates specified above. Duration of stay in a level 2 or level 3 critical care bed will be analysed using a mixed-effects negative binomial regression model, with a random intercept for centre. The model will adjust for the set of covariates specified above.

#### Subgroup analysis

We will conduct subgroup analysis of the primary outcome by urgency of surgery (immediate vs. urgent vs. expedited), age, gender, indication for surgery (bowel perforation vs. bowel obstruction without perforation vs. other indications), and NELA preoperative predicted risk score. We will also assess the impact of the COVID-19 pandemic on treatment effect by examining the following subgroups: (1) whether the participant was randomised pre- (< 30 January 2020) or post- (≥ 30 January 2020) COVID-19 pandemic and (2) COVID-19 status of participant (negative [0] or positive [1]), subject to test availability (March 2020 onwards).

Any subgroup findings will be treated with caution and will be given less weight than the primary analysis. Subgroup analyses will only include patients who have complete data for the primary outcome and for the subgroup variable of interest. For all subgroup analyses, the presence of an interaction will be assessed using a Wald test to simultaneously test whether all interaction terms in the model are non-zero. The test will be considered significant at the 5% level.

#### Exploratory analysis

We will explore the degree to which DAOH-90 can be used as a proxy for “days alive at home” within 90 days (DAH-90) by repeating the primary analysis using DAH-90. This will be conducted in the subset of participants with available information on discharge destination, recorded in NELA until December 2019. See Additional file 1 for full details.

### Health economic analysis

The economic evaluation will follow the NICE health technology evaluations manual [39] to ensure that trial findings are informative for national-level policy considerations. The perspective will be limited to NHS secondary care and the analysis will include the costs of the index hospital admission (including haemodynamic therapy) and subsequent hospital (re)admissions during 90 days and, separately, during 1 year from randomisation. NELA/eCRF will provide individual-level resource use information related to the primary hospital admission, including the haemodynamic therapies used and duration of stay in level 2/3 critical care. Routinely collected hospital care data (including inpatient and critical care episodes) will be obtained from NHS Digital (or devolved nation equivalents) for the post-randomisation periods to estimate total hospital care cost during 90 days and, separately, during 1-year follow-up periods from randomisation. Unit costs for individual resource use items will be informed from national sources (such as the NHS Reference Costs) where available; further sources will be used if necessary. These will be applied to individual patient resource use to evaluate patient costs.

Due to the lack of direct assessments of patients’quality of life in the trial, quality-adjusted life years (QALYs) will be evaluated using the EQ-5D-3L data in the EPOCH trial [4] and mortality data in FLO-ELA. This will involve estimating an EQ-5D-3L tariff prediction model in the EPOCH data using relevant patient characteristics common to patients in EPOCH and FLO-ELA, and applying that model to FLO-ELA patient data to predict EQ-5D-3L tariff values for participants in FLO-ELA during follow-up in the study.

The comparison of resulting QALYs and costs between treatment groups will broadly follow the primary outcome analysis (e.g. intention-to-treat basis, adjustment for minimisation factors and other pre-specified covariates). The economic analysis will be a cost-effectiveness analysis of cardiac output-guided fluid therapy compared to current usual care at 90 days and, separately, at 1 year combining survival within 90 days or, respectively, 1 year post-randomisation, with quality of life and costs of participants during this period. The cost-effectiveness analysis will be presented in terms of incremental costs per QALY gained at 90 days and, separately, 1 year post-randomisation. Incremental cost per death avoided at 90 days and, separately, 1 year post-randomisation will be also presented.

Uncertainty around cost-effectiveness results will be analysed using a bootstrap approach to evaluate the impact of joint uncertainty in QALYs and costs on cost-effectiveness. Cost-effectiveness acceptability curves (CEACs) and net benefits will be reported for willingness to pay values ranging from zero to £30,000 per QALY. Sensitivity analyses will be used to examine the effect of key assumptions on the results of the cost-effectiveness analyses.

To further guide policy, longer-term extrapolation will be considered in case of remaining policy uncertainty (e.g. evidence for survival benefit but unclear cost-effectiveness within 1 year post-randomisation), to project survival of participants and their healthcare costs and evaluate the cost-effectiveness of cardiac output-guided fluid therapy over a longer time period. A health economics analysis plan, specifying the health economic analyses in detail, will be finalised and signed off prior to unblinding the team analysing the study. Changes to the original health economic analysis plan are included in Additional file 1.

### Monitoring and audit: safety and data

All interventions within the FLO-ELA trial are already in routine clinical use for patients undergoing major gastrointestinal surgery. Serious adverse events (defined as an adverse event resulting in death, threat to life, hospitalisation [or prolongation of hospitalisation], or persistent disability/incapacity) which are judged to be related to the use of study procedures, and not an expected occurrence after abdominal surgery, will be reported.

The Sponsor and trials unit will have oversight of the trial conduct at each site. The Trial Management Group (TMG) is comprised of the Chief Investigator, senior co-investigators, support staff, and trials unit representatives. It will have day-to-day responsibility for safety reporting, quality control, and quality assurance of the data collected. Data completeness and protocol compliance will be monitored centrally by the TMG, with on-site study auditing performed by the trials unit in response to any data and governance issues raised.

An independent Data Monitoring and Ethics Committee (DMEC) and a Trial Steering Committee (TSC) have been appointed and function in accordance with NIHR guidance and an agreed charter (see Additional file 1 for full composition; charters available at www.floela.org/Study-Documents). No formal interim analysis for efficacy is planned. The DMEC will monitor the safety and efficacy of the interventions during the period of recruitment into the trial, and review patient recruitment, data quality, protocol compliance, and loss to follow-up. The DMEC will make recommendations to the TSC who will make final decisions on trial continuation.

### Patient and public involvement

Improving patient care in emergency surgery was a top ten research priority from the 2015 James Lind Alliance Priority Setting Partnership (Anaesthesia) between clinicians, patients, and the public [40]. Patient/public involvement started at the earliest stages of trial design, with input from patients with experience of emergency laparotomy and intensive care. The proposal was reviewed by the Royal College of Anaesthetists Patient, Carer and Public Involvement and Engagement (RCoA PCPIE) in Research Group. These discussions informed the trial design, particularly in developing a recruitment and consent process felt to be effective and acceptable to participants and their carers, including situations in which the patient lacks capacity. The trial group includes a lay co-applicant who sits on the trial steering group along with an independent lay member, advising on all aspects of trial decisions and conduct. A lay summary of the trial results will be made available to participants. Lay members of the FLO-ELA study group and the PCPIE group will facilitate dissemination to patient groups (e.g. bowel cancer, inflammatory bowel disease, intensive care) through focus groups and online media.

### Ethics and dissemination

The FLO-ELA trial has been approved by the UK National Research Ethics Service (NRES, IRAS 214459). All participating centres have full ethical approval. Any proposed major protocol amendments will require Sponsor and NRES approval, after which they will be disseminated to participating sites, funder, and ISRCTN public trial registry. The Sponsor will provide no-fault insurance.

Information related to participants will be kept confidential and managed in accordance with the Data Protection Act (UK), NHS Caldicott Principles (UK), The Research Governance Framework for Health and Social Care (UK), and the conditions of Research Ethics Committee Approval, or corresponding legislation or approvals for a particular participating site. The patient’s NHS/CHI number, gender, date of birth, and postcode will be collected at randomisation to allow tracing through national records. The personal data recorded on all documents will be regarded as confidential. Submitted data will be stored securely against unauthorised manipulation and accidental loss since only authorised users at site, the Sponsor organisation, Queen Mary University of London, or NELA (host of the data entry portal) will have access. Storage and handling of confidential trial data and documents will be in accordance with the Data Protection Act 2018 (UK) and General Data Protection Regulation.

Results arising from this research will be made available to the scientific community in a timely and responsible manner. A detailed scientific report will be submitted to a widely accessible scientific journal on behalf of the FLO-ELA Trial Group. Further dissemination will include presentations at international scientific meetings, public presentations, webcasts, and reports targeting international healthcare policy-makers, professional organisations, frontline healthcare workers, patients, and the public.

## Discussion

A number of changes have been made to the original protocol. The internal pilot showed that participant representativeness and protocol compliance satisfied the progression criteria (see Additional file 1). The number of recruiting sites and randomised participants were below target. Protocol version 2.0 included changes to include recruiting sites in Scotland and Northern Ireland and adapted the recruitment pathway to allow greater inclusion of participants not capable of giving prospective consent. The COVID-19 pandemic occurred during an ongoing review of trial recruitment rates and forced a pause in recruitment from 20 March 2019 to 7 September 2019. At the point of pausing recruitment, 2038 participants had been recruited. At the funder’s request, a trial recovery proposal was submitted. In November 2021, we introduced a modified primary outcome and sample size. The change from the originally planned primary outcome (mortality at 90 days after randomisation) and sample size (7646) was made with the approval of the TSC and trial funder. No members of the trial team, TSC, or funding body had access to, or knowledge of, ongoing trial results at the time the decision to modify the primary outcome was made. The new sample size calculation was made without knowledge of unblinded data. These changes were made in the protocol and SAP version 3.0. The current protocol and SAP version 4.0 introduce the estimand framework to the statistical analysis and add exploratory analyses of the impact of COVID-19 on the effectiveness of the intervention and wider care delivery in the trial. See Additional file 1. The trial protocol and SAP are available at www.floela.org/Study-Documents.

During the recruitment pause caused by COVID-19, the trial randomisation system was changed from the original system created by the trials unit to one provided by an external provider (sealedenvelope.com). This was a precautionary measure taken in response to an internal investigation that showed a minor imbalance in the random component of the minimisation algorithm. Both groups already randomised by the original system remained balanced in terms of numbers and characteristics, and allocation concealment had not been affected. Existing participant numbers within each minimisation factor were uploaded to the external system to allow accurate ongoing minimisation when recruitment restarted.

This trial has a number of strengths and limitations. It will be the largest contemporary randomised trial examining the effectiveness of perioperative cardiac output-guided haemodynamic therapy in patients undergoing major emergency gastrointestinal surgery. Days alive and out of hospital within 90 days of surgery, a composite outcome encompassing the effects of postoperative morbidity and mortality, is of clear importance to patients and healthcare systems. The multi-centre design and broad inclusion criteria support the external validity of the trial. The trial has an efficient and cost-effective design, harnessing existing datasets for data collection and clinician support for trial intervention delivery. Although the clinical teams delivering the trial interventions will not be blinded, significant trial outcome measures are objective and not subject to detection bias. The trial findings have the potential to impact care for tens of thousands of patients each year in the UK alone.

## Trial status

Participant recruitment began in September 2017 and is expected to complete by 31 December 2023. The current protocol is 4.0 (dated 27/04/2022).

## Supplementary Information

**Additional file 1.**

**Additional file 2.** SPIRIT checklist.

## Data Availability

At the point of recruitment to the trial, participants are invited to give consent for the onward sharing of anonymised data for further research by authenticated researchers who guarantee to preserve the confidentiality of the information requested. Requests for data sharing will be considered by the data sharing committee of the supporting trials unit (Pragmatic Clinical Trials Unit, Queen Mary University of London) in accordance with their data sharing policy. Any proposed data share will also be subject to the terms and conditions of the data sharing agreements already in place between the trial Sponsor and external data controllers (HQIP and NHS Digital) so we cannot guarantee that all requests will be satisfied.
