# Supplementary material for: Fluid Optimisation in Emergency Laparotomy (FLO-ELA) Trial: study protocol for a multi-centre randomised trial of cardiac output-guided fluid therapy compared to usual care in patients undergoing major emergency gastrointestinal surgery
Source: Trials. 2023 May 6;24:313. doi: 10.1186/s13063-023-07275-3 (PMC10163929; doi:10.1186/s13063-023-07275-3)
Supplement: Supplementary file 1 — Additional file 1. [file 13063_2023_7275_MOESM1_ESM.pdf]

Fluid Optimisation in Emergency Laparotomy (FLO-ELA) Trial: study protocol for a multi-centre trial of cardiac output-guided fluid therapy compared to usual care in patients undergoing major emergency gastrointestinal surgery: study protocol supplementary file.

Mark R Edwards, Gordon Forbes, Neil Walker, Dion G Morton, Monty G Mythen, Dave Murray, Iain Anderson, Borislava Mihaylova, Ann Thomson, Matt Taylor, Marianne Hollyman, Rachel Phillips, Keith Young, Brennan C Kahan, Rupert M Pearse, Michael P W Grocott for the FLO-ELA investigators.

Contents

1. FLO-ELA Trial Investigators 2
2. Oversight committee composition 7
3. National Emergency Laparotomy Audit (NELA) Inclusion/Exclusion criteria 8
4. Enrolment and recruitment to the FLO-ELA trial 10
5. Recommended intra-venous fluids 12
6. Cardiac Output Monitors 13
7. Definitions of levels of care 13
8. Baseline and Follow-Up Data 13
9. Changes to statistical analysis plan 39
10. Days At Home exploratory analysis 41
11. Changes to health economic analysis plan 41
12. Internal pilot phase results 41
13. Supplementary File References 43

1. FLO-ELA Trial Investigators

PI: Principal Investigator; API: NIHR Associate Principal Investigator

Southampton General Hospital: Dr Alexandra Skubala (Current PI), Dr Patrick Tapley (PI), Dr Suzanne Kellett (PI), Clare Bolger, Rachel Burnish, Nikki Collings, Dr Andrew Cumpstey, Dr Matt Taylor, Dr Hannah Wong, Dr Vic Rehnberg, Dr Jessica Lees, Karen Salmon, Dr Naomi Wee, Dr Sarah Harrison, Dr Li Ping Gan, Dr Claire Halloran, Dr Georgios Tsiopanis, Dr Said Seifalian, Dr Richard Webster, Dr Martin Knight, Dr Hannah Theobald, Dr Anna Clark, Dr Thomas Nicholls, Mr James Willey, Dr Sophia Beeby, Dr Luke Bracegirdle, Dr Kate Stoddard, Belinda Roberts, Alice Baker, Norma Diaper, Jonathan Biss, Michael Carter, Dr Francesca Riccio, Dr James Green, Lucy Johnstone, Jade Rand, Kasia Wisniewska, Grant Gibson, Hannah Bateson, Michelle Beveridge, Martyna Marani, Isabel Monger, Agnieszka Burtt, Sue Jackson, Dr Tim Prescott, Dr William Smith, Anne Harrison

Derriford Hospital: Dr Gary Minto (Current PI), Dr Iain Christie (PI), Dr Anna Fergusson (API), Abigail Patrick, Dr Stuart Cleland, Charlotte Eglinton, Natasha Wilmshurst, Fiona Reed, Joanne Smith, Dr Anna Ratcliffe, Dr Elizabeth Freeman, Jennie Kingdon, Dr James Humphreys, Abigail Patrick, Dr Sarah Nelson

Russells Hall Hospital: Dr Adrian Jennings (PI), Angela Watts, Andrew Moores, Lucy Smith, Dr Jenny Wright, Dr Julian Sonksen, Dr Caroline Moody, Dr Philip Harrington, Dr Jack Lee, Dr Nadim Kozman, Dr Zoe Riddell, Dr Catherine Brennan, Dr Shakira Nathoo, Dr Vikram Anumakonda, Dr Andrea Gait, Dr Richard Pierson, Dr Raj Patel, Dr Lee Plant, Dr Nipun Agarwal, Dr Hadassah Ihlenfeldt, Dr James Heggie, Dr Rachel Olive, Dr Joseph Pick, Sally Hinsley, Dr Nicola Calthorpe, Julie Matthews, Wendy Gardner, Dr Charlotte Topham, Dr Edward Jones, Dr Elliot Yates, Dr Sachin Sekhsaria, Dr Mohamed Amer, Dr Phil Pemberton, Dr Nicholas Coffin, Dr Halden Hutchinson-Bazely, Karen June Pearson, Tracy Edwards, Dr Beth Fitzmaurice, Dr Anna Pierson, Dr Katie Archer, Dr Omar Ahmed, Dr Sajid Khan Mohammed, Dr Alex Hollis, Dr Stephanie Weedon, Dr David Hillier, Dr Joanna Lau, Dr Vishal Amin, Dr Laura Dixon, Dr Joseph Seager, Dr Joe Tyler, Stacey Forsey, Dr N. Parry, Dr Aamer Mughal, Dr Jialuen Goh, Dr Rose Tiller, Dr Daniel Taylor, Dr Hasini Rallage, Dr Alexandra Leech

Leighton Hospital: Dr John Harris (PI), Claire Gabriel, Sheron Clarke, Katherine Pagett, Dr Thomas Rudnick, Nick Brown

Medway Maritime: Dr Sarah Hare (PI), Dr Eimhear Lusby (API), Dr Edward Bayliss (API), Dr Christopher Ward (API), Dr Rahul Bandopadhyay (API), Dr Kerrie Wilson, Dr Theodore Floyd, Iram Ahmed, Tom Hatton, Dr Malgorzata Szeszo, Thyra Kyere-Diabour, Dr Daniel Sumner, Tessa Lawrence, Emma Sutton, Dr Winston Ng, Dr Ioannis Kapsokalyuas, Dr Anthony Carter, Dr Anamika Kansal, Dr Leon Bernard, Dr Siew-Ling Harrison, Dr Andrew Feneley, Dr Owen Cooke, Dr Jennifer Hawley, Dr Sophie Berry, Laura Adams, Dr Thomas Hansen

James Paget Hospital: Dr Pieter Bothma (PI), Julie North, Teresa Ferreira, Dr Karan Verma, Dr Karthik Surendran, Dr Aruthy Arumugam

Lister Hospital: Dr Sunil Jamadarkhana (PI), Carina Cruz, Pearl Baker, Naomi Brice

Basingstoke and North Hampshire Hospital: Dr Antony Ashton (PI), McDonald Mupudzi, Dr Juliette Kemp, Ajay Rahl, Denise Griffin, Dr Aaron Stokes, Dr Keith Ritchie, Dr Arcot Venkatasubramaniam, Robert Cheek, Madonna Brown, Dawn Trodd, Caroline Wrey Brown, Jane Martin, Sam Hammond, Louisa Mason, Nycola Muchenje

Great Western Hospital: Dr Hamish Breach (PI), Amanda Colston, Dr Malcolm Watters, Dr Edwards Miles, Emma Marshall, Dr Madeleine Storey, Dr Victoria Hawley, Dr Edward Gomm, Dr Claire Potter, Melanie Knowles, Dr Edward Beech, Dr Peter van Breda, Helen Langton, Dr Nicholas Suarez, Dr Matthew Rowe, Mr Andrei Tanase, Dr Jonathan Barnes

Harrogate District Hospital: Dr David Earl (PI), Lorraine Stephenson, Tracy Burdett, Dr Martin Huntley, Dr Emma Cottrell, Dr Hao Ern Tan

Birmingham Heartlands Hospital: Dr Joyce Yeung (PI), Dr Jasraj Kailey, Teresa Melody, Jo Gresty, Julia Sampson, Katie Atterbury, Peter Sutton, Natalie Carling, Eleanor Reeves, Dr Carl Groves, Dr Daniel Crossmann, Sarah Ballinger, Rachel Smith, Marie Thomas, Dr Will Rook, Mohamed Mooradun, Dr Qasim Khan, Dr Arif Qureshi, Dr Llewellyn Fenton-May, Dr Adam Boulton, Dr Daniel Whitney, Dr Shilpa Sannakki, Dr Manekar Avinash, Dr Nikiesha Lee, Dr Neha Sharma

Kings Mill Hospital: Dr Srinivas Magham (Current PI), Dr Gareth Moncaster (PI), Rebecca Boulton, Terri-Ann Sewell, Wayne Lovegrove, Dr John Tansley, Mr Nick Watson, Sarah Shelton, Cheryl Heeley, Philip Buckley, Katie Slack, Rebecca Holmes, Andrea Palfreman, Christopher Smith, Mandy Gill, Sue Smith, Tracy Brear, Jill Kirk, Megan Holmes, Camelia Goodwin, Margaret Flynn, Inez Wynter, Kaytie Bennett, Emily Omuwie, Rachel Johnson, Jennifer Evans, Rebecca Rudd

Musgrove Park Hospital: Dr Stephen Harris (PI), Corrine Pawley, Patricia Doble, Moira Tait, Dr Richard Gibbs, Miss Marianne Hollyman, Mr Tom Edwards, Mr Paul Mackey, Miss Louise Hunt, Jo Hutter, Mr Ed Smyth, Mr Hamish Noble, Dr Thomas Judd , Dr Rose Arkell, Dr Owen Thomas, Dr Karen Watura, Dr Marius Vaida

Royal Free Hospital: Dr Suehana Rahman (PI), Dr Saaidullah Sufi (API), Helder Filipe, Christine Eastgate, Margaret McNeil, Stephen Howey, Glykeria Pakou, Sara Mingo, Amitaa Maharajh, Dr Isobel Loeffler, Catarina Veiga, Poh Choo Teoh

Royal Hampshire County Hospital: Dr Irina Grecu (PI), Dr Martin Knight (API), Dawn Trodd, Samantha Hammond, Caroline Wrey Brown, Jane Martin, Dr Susan Hanson, Dr Patrick Tapley, Dr Julia Ottaway, Dr Victoria Burgess, Dr James Fry, Dr Geoff Watson, McDonald Mupudzi, Dr Francois Wessels, Denise Griffin, Dr Hugh Cutler, Dr Arthur Goldsmith

Sandwell General Hospital: Dr Mark Howes (Current PI), Dr Subash Sivasubramaniam (PI), Julie Colley, Jenny Porter, Dr K.P. Krishnan, Dr Kerrie Aldridge, Sylvia Willetts, Carol Zullo, Mr Christopher Thompson

Stoke Mandeville Hospital: Dr Pradeep Shanmugasundaram (PI), Judith Abrams, Katarina Manso, Jamil Razzaque, Sally Scott, Geraldine Hambrook, Elizabeth McKerrow, Tahir Ali, Alastair Ankers

Warwick Hospital: Dr Mohan Ranganathan (PI), Dr Sunil Bellam, Sophie Mason, Mr Paul Marriott, Dr Richard Robley, Bridget Campbell, Penny Parsons, Mr Sean Ramcharan, Dr Susanne Mohamed Anver, Dr Samuel Chambers, Dr Oleg Bumbac

Watford General Hospital: Dr Valerie Page (PI), Elaine Walker, Xiaobei Zhao, Dr Laura Osborne, Dr Beena Parker, Dr Rupinder Kaur, Dr Gitana Kulakauskaite-Rasteniene, Dr Mehul Patel

Conquest Hospital: Dr Alastair Lowe (PI), Emma Edmunds, Dr Kieran Hills, Dr Michail Klimovskij, Dr Christopher Ranns, Dr Radha Ganesh, Dr David Jones, Dr Jamie Gibson, Janet Sinclair, Karen Burton, Toni De Freitas, Dr Leon Dryden, Dr Amelia Robinson, Dr Nnamdi Udezue, Dr Tim Faccini, Dr Moon-Moon Majumdar, Kelly Death, Dr Hide Baba, Dr Jaydraman Narendran, Dr Ross Holcombe-Law, Dr Alexandria Page, Dr Paul Jackson

Bradford Royal Infirmary: Dr Bret Claxton (Current PI), Dr Andrew Brennan (PI), Louise Akeroyd, Dr Sarah Cooper, Dr Catherine Farrow, Dr Carl Ilyas, Dr James Morgan, Dr Craig Montgomery, Dr Brian Wilkinson, Dr John Dereix, Dr Karen Eaves-Lai, Dr Kate Pye, Dr David Craske, Dr Paul Panesar, Dr Peter Hart, Dr Mark Stubbington, Kim Storton, Kelvin Stewart, Dr Laura Graham, Shereen Bano, Dr Robert Neal, Declan Ryan-Wakeling, Dr Varun Chauhan

St Thomas' Hospital: Dr Michael Shaw (PI), Maame Aduse-Poku, Soo Jin Kang, Gill Arbane, Dr Kariem El Boghdadly, Dr Paul Kelly, Dr Jaimin Patel, Dr Marcin Sicinski, Dr Martin John, Dr Mark Ibrahim, Dr Emad Aziz, Dr Sohini Sengupta, Dr Melissa Baldwin, Dr Toby Dixson, Dr Leslie D'souza, Dr Charlotte Taylor, Dr Suneil Rameseur, Dr Heena Bidd, Dr Guy Glover, Dr Andrew Morley, Dr Asta Lukosiute, Dr Anna Janowicz, Dr Tamara Alexander, Dr Melissa Baldwin, Dr Joe Lipton, Dr Rathai Anandanadan, Dr Dale Seddon, Alexander Phillips, Dr Louise Davies, Dr Sanjoy Bhattacharyya, Dr Soo Yoon, Dr Sian Fraser, Dr Alex Stilwell, Dr Karin Shoeman, Dr Justin Hews, Dr Sivanth Sivakumar, Dr Floju Chin, Dr Declan Dudley, Gary Colville, Abegail Sawana, Dr Jakob Dudziak

Kingston Hospital: Dr Britta O'Carroll-Kuehn (PI), Dr Chandran Jeganathan (Current PI), Dr Nicole Richards, Andrew Swain, Charlotte Quamina

St James's University Hospital (Leeds): Dr Indu Sivanandan (Current PI), Dr Simon Whiteley (PI), Dr Paul Panesar (API), Elizabeth Wilby, Clare Howcroft, Charlotte Trumper, Kate Varley, Dr Sharmeen Lotia, Dr Eugene Henry, Dr Claire Schofield, Dr Ntima Ntima, Dr Omar Jundi, Dr Amelia Schorah, Dr Luke McMenamin, Dr Robert Jackson, Dr John Jones, Suzie Colquhoun, Dr Joana Faria, Nora Youngs, Dr Carl Ilyas, Dr Craig Montgomery, Aneesha Qadeer, Judith Sharp, Rosie Wragg, Michelle Naylor, Beverley Jackson, Catherine Moriarty, Louise White, Dr Laura Wade, Dr Brian White, Samuel Craven

Peterborough City Hospital: Dr Coralie Carle (PI), Alan Pope, Dr Mark Chen, Nicola Butterworth-Cowin

Queen Elizabeth Hospital Birmingham: Dr Rajneesh Sachdeva (PI), Karen Ellis, Colin Bergin, Emma Reeves, Amy Bamford, Maximina Ventura, Dr Tony Whitehouse, Ronald Carrera, Elaine Spruce, Liesl Despy, Samantha Harkett, Morgan Foster, Mr Antonios Athanasiou, Mr Kamran Malik, Stephanie Porter, Dr Jaimin Patel, Dr Randeep Mullhi, Craig Sadler, Dr James Gasbey, Christopher McGhee, Dr Jignesh Patel, Tracy Mason, Hazel Smith, Alexandra Efimov, Aoife Neal, Stephanie Goundry

The Royal London Hospital: Dr Davina Ross-Anderson (PI), Dr Kath MacGloin, Dr James Pennington, Tim Martin, Edyta Niebrzegowska, Dr Mevan Gooneratne, Dr Chhaya Sharma, Dr Neil MacDonald, Jan Whalley, Priyanthi Dias, Dr Gareth Ackland, Dr Peter Shirley, Tim Stephens, Dr Parjam Zolfaghari, Steven Dunkley, Dr Toby Reynolds, Dr Henry Walton, Dr Matthew Smith, Dr Shreya Bali, Sara Hui, Dr Ravi Bhatia, Dr Hew Torrance, Maria Fernandez, Ruzena Uddin, Filipa Santos, Amaia Arrieta, Dr Stephen Barrett, Dr Richard Cashmore, Dr Eleanor Catherine Richards, Fatima Seidu, Dr Edward McIlroy, Dr Thomas Urwin, Dr John Samuel, Dr Katherine Brooks, Dr Natalie Gravell, Dr Douglas Blackwood, Dr Tanj Sanghera, Mareena Joseph, Dr Aisha Jalaly, Dr Hannah Nugent, Dr Daniel George

Royal Victoria Infirmary: Dr Ben Goodman (PI), Dr Ashley Scott (API), Dr Ian Clement, Leigh Dunn, Abigail Harrison, Carole Hays, Maite Babio-Galan, Dr Sarah Todd, Dr Lindsay Dawson, Stephanie Henderson, Dr Kapil Arora, Dr Subramani Diwaker, Sam Marcangelo, Dr James Harvey, Dr Mark Dalton, Dr Jeremy Hyams, Tara Shrestha, Kimberley Zwiggelaar, Dr Laura Heggie, Dr Rhona Sinclair, Ben Brown, Tess Wilkinson

Sunderland Royal Hospital: Dr Sean Cope (PI), Dr Steven Traill (API), Dr James Durrand (API), Julie Sheriff, Ashley Allan, Lindsey Woods, Dr Erin Chuter, Rebecca Betts, Dr Rossalyn Thistlethwaite, Dr Elizabeth Turnbull, Dr Monojit Paul, Dr Pete Campbell, Dr Vlad Bashlijski, Dr Charlotte Foley, Dr Amy Ginn, Dr Adam Cookson, Sarah Cornell, Dr Janaki Pearson, Kimberley Rogerson,

University Hospital Lewisham: Dr Ben Eden-Green (Current PI), Dr Marthin Mostert (PI), Dr Maria Leong (API), Dr Daniel James Kirkin (API), Rosie Reece-Anthony, Dr Fatima Ali, Dr Michaela Heller, Dr George Mathew, Dr Beenu Madhavan, Dr Katherine Powell, Dr Noelene Dasey, Dr Waqas Khaliq, Babita Gurung, Dr Cristina Alkhadra, Dr Tarek Mostafa, Dr James Winchester, Dr Megan Thomas, Dr Amit Soodan, Alfa Cresia N. Nilo, Dr Daniel Kirkin

Aintree University Hospital: Dr Matthew Bridge (PI), Colette Jones-Criddle, Laura Wilding, Ian Turner-Bone, Dr Ben Morton, Mr Tim Gilbert, Dr Nathan Littley, Dr Natasha Clarke, Dr Greg Moore, Dr Tamryn Miller, Dr Tom Rudnick, Dr Tom Miller, Michaela Lloyd, Leanne Smith, Amie Reddy, William McCaig, Harriet Murrant

Queen's Hospital – Burton: Dr Manab Haldar (Current PI), Dr Ashok Nair (PI), Dr Michael Jarvis (API), Precious Basvi, Gillian Bell, Michelle Edwards, Clare Mewies, Mr Stelios Vakis, Dr Emily Burton, Dr Kiran Yelamati, Dr Amit Das, Dr Abhimanyu Bhattacharya, Dr Daniel Massey, Dr Ramkumar Kalaiyarasan, Dr Amro Katary, Dr Win Maung, Dr Dave Robinson, Dr Beth Frost, Dr Samuel Besant, Dr Sunita Gurung, Dr Agah Isguzar, Dr Mina Amirhom, Dr Muhammad Javid, Dr Ammara Masoud, Dr Mia Davis, Dr Debasis Pradhan, Joanne Rudkin, Dr Matthew Byrne

Croydon University Hospital: Dr Ashok Raj (PI), Dr Gabrielle Adkins (API), Dr Rahim Nadeem Ahmed, Dr Josphine Cashman, Dr Elizabeth Smee, Dr Clare Ivermee, Dr Charlotte Cobain, Dr Rahim Nadeem Ahmed, Dr Ana Robles, Dr Yin Choo, Ms Reena Khade, Dr Georgios Tsinaslaniois, Dr Vasileios Bafitis, Dr Christopher Black, Cassandra George

University Hospital Of North Tees: Dr Narayanan Suresh (PI), Lynne Williams, Michele Clark, Pam Race, Dr Anil Agarwal, Dr James Durrand, Dr Rakesh Bhandary, Dr Valasubramaniam Mahadevan, Dr Monojit Paul, Dr Michael Courtney

The Royal Bournemouth Hospital: Dr James Walker (PI), Dr Susan Hayward, Luke Vamplew, Sally Pitts, Debbie Branney, Andrew Bates, Katie Molloy, Nina Barratt, Dr Sarah Turle, Dr Doug Tunney, Dr Erica Jolly, Tallulah Webb, Katie Bowman, Jessica Kelly, Rebecca Miln, Marketa Keltos, Maria Letts

Royal Cornwall Hospital: Dr Juan Graterol (PI), Fiona Hammonds, Jessica Summers, Belinda Wroath, Gabbie Young, Benita Adams, Nicki Devooght-Johnson, Eve Fletcher, Michele Wall, Kelly O'Toole, Dr Allwyn Cota, Dr Richard Hunt, Toby Nisbett, Dr Sam Spinney

Royal Derby Hospital: Dr Tanuja Shah (PI), Dr Brett Doleman (API), Dr Laura Carrick, Dr C.Nagendra Prasad, Dr Kathleen Holding, Lianne Hufton, Mr William Speake, Mr Philip Herod, Dr James Nayyar, Dr Daniel Stolady, Shuing Wei, David Daly, Corinne Paxton

Southport District General Hospital: Dr Tauseef Ahmed (Current PI), Dr Anwar ul Huda (PI), Dr Christopher Goddard, Mr Paul Ainsworth, Dr Thomas James Murphy, Barry Jones, Anna Morris, Helen Terrett, Dr John Kirby, Dr Ann Holden, Dr Mark Spiliopoulos, Dr James Hammond

University Hospital of North Durham: Dr Iain Cummings (Current PI), Dr Helen Melsom (PI), Louise Duncan, Dr Sameer Somanath, Andrea Kay, Melanie Kent, Michelle Wood, Sarah Clark, Mr Liam O'Hare, Dr Lewis Schofield, Ami Laidlaw, Dr Jordan Minns, Dr James Roe, Dr Charlotte Foley, Stefanie Hobson, Suzanne Naylor, Vicki Atkinson, Victoria Allinson

Bristol Royal Infirmary: Dr Phoebe Syme (PI), Lisa Grimmer, Kate Driver, Libby Cole, Denise Webster, Dr John Hickman, Dr Carly Webb, Katie Sweet, Dr Edward Mew, Dr Sarah Warwicker, Dr Susan Tetlow, Dr Alex Middleton, Dr Jonathan Rees, Dr Chris Gough, Dr Sam Howell, Chloe Searles, Dr Shelley Barnes, Dr Louise Seller, Dr Jim Dunham, Dr Alastair Brown, Zoe Garland, Dr Adam Duffen, Dr Jim Dunham, Dr Thomas Renninson, Anna Chillingworth, Dr Paul Watson, Dr Alex Jones, Rebekah Johnson, Dr Krisztina Kenesey, Dr Thomas Cope, Dr Samuel Fitzpatrick, Dr Annie Amphlett, Dr Christopher Sajoler, Dr James Matthams, Dr Natalie Constable, Dr Jo Poole, Dr Hannah Wilson, Dr Liz Hood, Dr Ruth Greer, Dr James Self, Dr Katherine Nickell, Dr William Headdon, Dr Charlotte Earnshaw, Dr Katie Samuel

Glan Clwyd Hospital: Dr Richard Pugh (PI), Jill Andrews, Sarah Evans, Dr Zain Habib, Dr Claudia Variu, Dr Mohammed Zardab, Dr Amy Ellison, Victoria Garvey, Dr Richard Morgan, Dr Shobna Ramakrishnan, Dr Shrisha Shenoy, Dr Michael Kriger, Dr Hefin Llewellyn, Dr Sophie Horrocks, Dr Sam McBride, Rachel Mawley, Dr Alexander Pereiradelima

Royal United Hospital (Bath): Prof Tim Cook (PI), Dr Emira Kursumovic (API), Sarah Hierons, Lucy Howie, Dr Stuart Younie, Lidia Ramos, Dr Tom Cloke, Dr Sara-Catrin Cook, Dr Ben Savage, Dr Alex Dunn, Dr Mark Sheils, Sarah Mitchard, Dr Matthew Gibbins, Dr Olivia Cheetham, Dr Neil Choudhuri, Dr Amelia Davies, Dr Peter Steed, Dr Abigail Harper, Dr Dipayan Choudhuri, Sarah Mitchard, Dr Ben Ballisat, Carrie Demetriou, Dr Tim Cominos, Dr Paul Watson, Dr Rebecca Powell, Gabrielle Evans

Torbay Hospital: Dr Johannes Retief (Current PI), Dr Thomas Clark (PI), Dr Jane Montgomery (PI), Dr Rachel Remnant (API), Dr Ciska Uys (API), Gabrielle de Selincourt, Dr Sally Ward-Booth, Dr Simon George, Dr Omar Islam, Dr Adam Revill, Dr Daniel Paul, Dr David Portch, Pauline Mercer, Elaine Vandecandelaere, Lorraine Thornton, Dr Victoria Field, Ken Almedilla, Dr Natalie Smith, Dr Jennifer Moran, Dr Thomas Hunt, Dr James Womersley, Raine Thornton, Anne McCarthy, Fleur Rogers, Julie Merizouris, Dr Will Hare, Dr Jonathan Carter, Dr Katie Flower, Dr Greg Warren, , Dr Ben Whatley, Dr Virginia Francis, Julie Wollaston, Alex Redome, Dr Louise Cossey, Dr William Spencer, Dr Mike McGovern, Dr Vicky Lewis, Dr Matthew Boyd

Southmead Hospital: Dr Christopher Newell (PI), Dr Sethina Watson (API), Beverley Faulkner, Emma Gendall, Kati Hayes, Ruth Worner, Elizabeth Goff, Dr Tim Howes, Dr Ben Ballisat, Dr David Cronin, Dr Jacques Carver, Dr Edward Mew, Dr Daragh Lehane, Dr Kath Jenkins, Dr Matthew Townsend, Dr Ruth Greer, Dr Helen Williams, Dr Gemma Nickols, Jodie Garrett, Dr Thomas Cope, Dr Alexander Jones, Dr Benjamin Savage, Dr Swati Gupta, Dr Mark Dorrance, Dr Edward Lent, Kerry Smith, Dr Dragos Dragnea, Dr Rebecca Williams, Dr Shelley Barnes, Dr James Self, Dr Emma Jenkins, Dr Ben Whatley, Dr Richard Mason, Dr Lydia Osborne, Dr Matt Thomas

Yeovil District Hospital: Dr Agnieszka Kubisz-Pudelko (PI), Dr Mohamed Gheith, Joanna Allison, Alison Lewis, Kate Beesley, Lucy Pippard, Dr Andrew Shrimpton, Tressy Pitt-Kerby, Dr Jeremy Reid, Nigel Beer, Jess Perry

King's College Hospital - Denmark Hill: Dr Matthew Garner (PI), Harriet Noble, Sian Saha, Eleanor Corcoran, John Smith, Evita Pappa, Louise Greig, Dr James Bland, Dr Gudrun Kunst, Dr Elena Stanton, Dr Owen Cooke, Emma Clarey, Dr Joe Macmillan

Northwick Park/ St Marks Hospital: Dr Tamsin Rope (PI), Dr John Shenouda (API), Dr Thomas O'Dell, Dr Hannah Matthews, Temi Adedoyin, Dr Nicola Schunke, Dr Rebecca Mersh, Dr Rosie May, Dr Ashraf Mohammed, Dr Cara Lewis, Dr Eoin Harty, Dr Jonny R Stephens, Dr Abigail Richardson, Dr Roger Sharpe, Dr Chima Oti, , Dr Palitha Bopitiya, Mr James

Read, Dr Kiran Chima, Dr Maria Henriksson, Dr Alexa Prichard, Fei Long, Dr John Brandreth, Dr Puvan Suppiah, Winnie Anunda, Dr Kathryn Singh, Dr Claire Ruck, Dr Eleanor Roderick, Dr Isabelle Kamenou, Najwa Soussi

St Helier Hospital: Dr Putul Sarkar (Current PI), Dr Stefan Wiebe (PI), Dr Yadullah Syed, Dr Aishi Lim, Kerry Barnes, Dr Kwabena Mensah, Dr Aidan Fullbrook, Dr Larry Mulleague, Dr Matt Varier, Dr Viplaw Shukla, Dr Ravi Srinivasan, Dr Najam Pervez, Dr Natasha Schneider, Dr Jasmin Shahnavaz, Dr Duncan Bailey, Dr Charlotte Cobain, Rosavic Chicano, Neringa Vilimiene, Dr Shahzaib Ahmad

William Harvey Hospital: Dr Neil Richardson (PI), Dr Youssef Mahmoud (API), Dr Shanni McDonald, Natasha Schumacher, Dr Svetlana Velinova-Teron, Dr Rohit Silhi, Dr Guy Chivers, Julie-Ann Davies, Tracey Cosier, Dr Barry Featherstone, James Rand, Dr Esther Cook, Dr Diana Neely, Dr John Coombes, Dr Harpreet Sodhi, Dr Thomas Burr, Dr Mark Oliver, Dr Michelle Walters, Dr Kim Jemmett, Dr Claudia Dulea, Lucy Cooper, Dr Sam Mcferran, Dr Maxime Rigaudy, Dr Kim Jennett, Heather Weston, Reanne Solly, Emma Ignall, Cathy Praman, Dr Ayman Nash, Dr Prathiban Kumar

Nevill Hall Hospital: Dr Vincent Hamlyn (PI), Dr Gayathri Chinnappa-Srinivas, Angie Organ, Dr Tudor Vlad Moisin, Dr Julia Parnell, Dr Matthew Whitehead, Dr Olivia Hayward, Dr Rosie Malkin, Dr Zoe Bennetton, Dr Anne Devine, Dr Tim Green, Dr Joanna Hubert

Gloucestershire Royal Hospital: Dr Sam Andrews (PI), Deborah Ward, Pauline Brown, Nick Vallotton, Jon Glass, Susan O'Connell, Dr Alice Bevan, Dr Tom Brougham, Dr Lawrie Kidd, Dr Sian Hughes, Dr Jeannine Stone, Dr Deborah Mann, Dr Helen Murray, Dr Fiona Davis, Dr Mark Eveleigh, Dr Jake Hartford-Beynon, Dr Katherine Stratton, Dr Kat Yan Yee Ng, Dr Thomas Renninson

Queen's Hospital Romford: Dr Mandeep Phull (PI), Dr George Joseph, Dr Kalyani Gorrela, Dr Viraj Weerasekera, Dr Nazneen Sudhan, Dr Ayub Khan, Dr Louis Chalmers, Dr Ben Huntley, Dr Sandra Chipperfield, Aparna George, Lace Paulyn Rosario, Tatiana Pogreban, Dr Robert Buhain, Dr Alia Hussain, Dr Asya Veluso Costa, Dr Eleanor Richards

Royal Devon & Exeter Hospital: Dr Thomas Clark (Current PI), Dr Charles Gibson (PI), Dr Sandeep Kusre (PI), Melanie Hutchings, Sinéad Kelly, Dr Rebecca Pugsley, Dr Hamza Malik, Dr Alec Beaney, Dr Tom Woodward, Dr Zahra Essackjee, Dr Kangni Chen, Dr Bruce McCormick, Dr Cath Matthews, Michelle Walter, Elizabeth Gordon, Sadie Heddon, Dr Timothy Warrener, Dr Peter Valentine, Dr Joel Prescott, Samantha Keenan

Worcestershire Royal Hospital: Dr Emily Johnson (PI), Eleanor Higgs, Jessica Thrush, Dr Laura Tulloch, Dr Fiona Osborne, Dr Victoria Poyntz, Pooja Takhar, Connie Rowlands, Dr Michael Mcalindon, Dr Victoria Lacey, Dr Nicholas Cowley, Stephanie Chamberlain, Sally Rudge, Dr Sian Bhardwaj, Dr David Freeman, Dr Brendan Spooner, Dr Satinder Dalay, Dr Nick Fitton, Rebecca Davies, Kay Fisher, Dr Laura Naumann, Dr Elma Wong, Dr James Graham

Royal Shrewsbury Hospital: Dr Simon Leach (PI), Helen Moore, Rebecca Wilcox, Dr Rhys Parry, Alison Magness, Mandy Carnahan, Dr Matthew Travis, Colene Adams, Dr Samuel Passey, Dr Richard Colebrook, Dr David Elcock, Priscilla Mhembere, Jayne Rankin, Dr Yee Yin Cheng, Dr Chris Clulow, Jo Stickley, Anne Carter, Alison Stephens, Elizabeth Buckingham, Laura Price, Ryan Jones, Andy Taylor, Dr Andrew Donnaly

Norfolk & Norwich University Hospital: Dr Maria Ochoa-Ferraro (PI), Dr Harriette Beard (API), Dr Jeremy Corfe, Jocelyn Keshet-Price, Deidre Fottrell-Gould, Gill Foot, Lisa Hudig, Karen Convery, Martyn Oliver, Dr Hannah Neil, Georgina Randell, Dr Melanie Maxwell, Dr Kavitha Kuntumalla, Dr Pushpaj Gajendragadkar, Dr James Wu

University Hospital of Wales: Dr Danielle Huckle (PI), Dr Stephen Petley, Dr Nadine Jones, Karen Rahilly, Gail Williams, Dr Margaret Coakley, Laura Jones, Dr Dominic Manetta-Jones, Dr Sara Churchill, Dr Llewellyn Fenton-May, Martin Grigg

Whipps Cross Hospital: Dr Davina Ross-Anderson (PI), Dr Laura Fulton (Co-PI), Dr Suyogi Jigajinni (Co-PI), Dr Emma Collins (API), Tim Martin, Fillipa Santos, Dr Noah John, Dr Abhilash Das, Dr Christopher Manville, Dr Tom Abbott, Dr Hester Carter, Fatima Seidu, Dr Lina Kanapeckaite, Dr Gavin Stead, Dr Jonathan Holmes, Dr Amy Ireson, Dr Edward Gill, Dr Stephanie Kwok, Dr Ariana Singh, Dr Aastha Chawla, Dr Calum McGrady

Manchester Royal Infirmary: Dr Alastair Duncan (PI), Dr Hannah Greenlee (API), Ian Venables, Rose Jama

Queen's Medical Centre Nottingham: Dr Iain Moppett (PI), Cecilia Peters, Lucy Ryan, Louise Conner, Megan Meredith, Amy Clark, Dr Abi Noah, Dr Louise Potter, Dr Will Lindsay, Dr Jaina Parmar, Dr Dan Harvey, Dr David Evans, Dr Marc Chikhani, Dr Ben Lowe, Dr Kevin O'Donoghue, Dr Avninder Chana, Rachel Roke,

Weston General Hospital: Dr Ioannis Tsagurnis (PI), Harvey Dymond, Dr David Sleep, Kristina Owens, Dr Rachel Beer, Dawn Simmons, Donna Cotterill, Dr Ime Eka, Sandra Beech, Dr Pei Jean Ong, Dr David Ritchie, Susan Wilkinson, Dr Matthew Butler, Dr David Crossley, Dr Victoria Van Der Schyff, Dr Irum Ghazanfar, Dr Andrew Mawer

Cheltenham General: Dr Sam Andrews (PI), Ana Almeida, Lucy Duggal, Dr Jonathan Lightfoot, Dr Anna Simpson, Deborah Ward, Pauline Brown, Nick Vallotton, Jon Glass, Susan O'Connell, Dr Tom Rennison

Victoria Hospital, Fife – Dr Robert Thompson (PI), Susan Fowler, Sandra Pirie, Patricia Cochrane

Aberdeen Royal Infirmary – Dr David Nesvadba (PI), Dr Patrice Forget, Pauline Ganley, Jennifer Noble, Amanda Coutts, Dr Nicola Walker, Dr Christopher Brennan

2. Oversight committee composition

The oversight committees are independent from the sponsor and trial management team, and were appointed by the funder. All members must declare any potential conflicts of interest. All members have signed up to oversight committee charters defining working practices and responsibilities. These are available at www.floela.org/Study-Documents

2.1 Data Monitoring and Ethics Committee (DMEC)

Name	Role and Institution	DMEC role
Professor Ian Roberts	Professor of Epidemiology & Public Heath, London School of Hygiene and Tropical Medicine, London, UK	Chair
Dr Tim Morris	Principal Research Fellow in statistical methods, MRC Clinical Trials Unit, London, UK	Independent member
Professor Danny McAuley	Professor of Intensive Care Medicine, Northern Ireland Clinical Trials Unit, Belfast, UK	Independent member

2.2 Trial Steering Committee (TSC)

Name	Role and Institution	TSC role
Professor Stephen Brett	Professor of Critical Care, Hammersmith Hospital, London, UK	Chair
Professor Mark Peters	Professor of Paediatric Intensive Care, Great Ormond Street Hospital, London, UK	Independent member
Dr Andrew Klein	Consultant in Anaesthesia, Royal Papworth & Nuffield Hospitals, Cambridge, UK	Independent member

Dr Suzie Cro	Senior Lecturer in Medical Statistics & Clinical Trials, Imperial Clinical Trials Unit, London, UK	Independent member
Carol Green	N/A	Independent member (lay member)
Dr Mark Edwards	Consultant in Anaesthesia and Perioperative Medicine, Southampton, UK	Non-independent member
Professor Mike Grocott	Professor of Anaesthesia and Intensive Care Medicine, Southampon, UK	Non-independent member

3. National Emergency Laparotomy Audit (NELA) Inclusion/Exclusion criteria

version 1.7, dated 24/02/2017

NELA Inclusion Criteria

NELA will enrol the patients treated in England or Wales who meet the following criteria:

- aged 18 years and over
- who undergo an expedited, urgent or emergency (NCEPOD definitions) abdominal procedure on the gastrointestinal tract

This will include:

- Open, laparoscopic, or laparoscopically-assisted procedures
- Procedures involving the stomach, small or large bowel, or rectum for conditions such as perforation, ischaemia, abdominal abscess, bleeding or obstruction
- Washout/evacuation of intra-peritoneal abscess (unless due to appendicitis or cholecystitis - excluded, see below)
- Washout/evacuation of intra-peritoneal haematoma
- Bowel resection/repair due to incarcerated incisional, umbilical, inguinal and femoral hernias (but not hernia repair without bowel resection/repair) E.g. large incisional hernia repair with bowel resection.
- Bowel resection/repair due to obstructing/incarcerated incisional hernias provided the presentation and findings were acute. This will include large incisional hernia repair with division of adhesions.
- Laparotomy/laparoscopy with inoperable pathology (e.g. peritoneal/hepatic metastases) where the intention was to perform a definitive procedure. This does not include purely diagnostic procedures.
- Laparoscopic/open adhesiolysis
- Return to theatre for repair of substantial dehiscence of major abdominal wound (i.e. "burst abdomen")
- Any reoperation/return to theatre for complications of elective general/upper gastrointestinal surgery meeting the criteria above is included. Returns to theatre for complications following non-GI surgery are now excluded (see exclusion criteria below).

If multiple procedures are performed on different anatomical sites within the abdominal/pelvic cavity, the patient would be included if the major procedure is general surgical. E.g.

- Non-elective colonic resection with hysterectomy for a fistulating colonic cancer would be included as the bowel resection is the major procedure
- However bowel resection at the same time as emergency abdominal aortic aneurysm repair would not be included as the aneurysm repair is the major procedure

The above criteria are not exhaustive. The NELA team should be contacted if any clarification is required.

NELA Exclusion Criteria

Patients with the following characteristics will be excluded from NELA:

1. Patients under 18
2. Elective laparotomy / laparoscopy
3. Diagnostic laparotomy/laparoscopy where no subsequent procedure is performed (NB, if no procedure is performed because of inoperable pathology, then include)
4. Appendicectomy +/- drainage of localised collection unless the procedure is incidental to a non-elective procedure on the GI tract
5. Cholecystectomy +/- drainage of localised collection unless the procedure is incidental to a non-elective procedure on the GI tract (All surgery involving the appendix or gallbladder, including any surgery relating to complications such as abscess or bile leak is excluded. The only exception to this is if carried out as an incidental procedure to a more major procedure. We acknowledge that there might be extreme cases of peritoneal contamination, but total exclusion avoids subjective judgement calls about severity of contamination.)
6. Non-elective hernia repair without bowel resection or division of adhesions
7. Minor abdominal wound dehiscence unless this causes bowel complications requiring resection
8. Non-elective formation of a colostomy or ileostomy as either a trephine or a laparoscopic procedure (NB: if a midline laparotomy is performed, with the primary procedure being formation of a stoma then this should be included)
9. Vascular surgery, including abdominal aortic aneurysm repair
10. Caesarean section or obstetric laparotomies
11. Gynaecological laparotomy
12. Ruptured ectopic pregnancy, or pelvic abscesses due to pelvic inflammatory disease
13. Laparotomy/laparoscopy for pathology caused by blunt or penetrating trauma
14. All surgery relating to organ transplantation (including returns to theatre for any reason following transplant surgery)
15. Surgery relating to sclerosing peritonitis
16. Surgery for removal of dialysis catheters
17. Laparotomy/laparoscopy for oesophageal pathology
18. Laparotomy/laparoscopy for pathology of the spleen, renal tract, kidneys, liver, gall bladder and biliary tree, pancreas or urinary tract

Returns to theatre for complications (e.g. bowel injury, haematoma, collection) following non-GI surgery are now excluded i.e. returns to theatre following renal, urological, gynaecological, vascular, hepatic, pancreatic, splenic surgery are excluded.

The term “emergency” laparotomy is defined in line with NELA. The time-frames are based on the National Confidential Enquiry into Peri-Operative Deaths (NCEPOD) 2004 (1), to encompass the following categories: “immediate” surgery (required within two hours of the decision to operate), “urgent” surgery (required within 2-18 hours of the decision to operate) and “expedited” surgery (required within days of the decision to operate).

During the course of the trial the NELA Project Team may make minor modifications to the definitions of surgical cases included within the audit. In this circumstance the inclusion/exclusion criteria for FLO-ELA will be amended to ensure consistency with NELA. Although hospitals in Scotland/Northern Ireland (NI) are not participating in the NELA program, the same procedural eligibility criteria will be used.

4. Enrolment and recruitment to the FLO-ELA trial

Potential participants will be screened by clinical and research staff at the site having been identified from operating theatre lists and by communication with the relevant nursing and medical staff. Due to the randomisation at an individual level, participant consent will be sought. Prior data suggests a majority of eligible patients will have capacity to consent (2,3). However, this trial also seeks to include participants who are incapable of giving consent to enter the trial for a number of reasons:

1. Patients may be experiencing severe abdominal pain or vomiting and have received strong analgesia, or may require multiple medical interventions in the time available before surgery.

2. Patients with potentially life-threatening acute conditions may require surgery in an urgent time frame. During this limited time, priority must be given to medical information and consent for surgery.

3. Patients may lack mental capacity due to acute delirium, or sedation in an intensive care setting.

Furthermore, due to the unanticipated nature of this surgery, there would not be an opportunity to perform consent before admission to hospital. These patients are an established exception to the general rule of informed consent in clinical trials, in accordance with the Declaration of Helsinki 2013, the Mental Capacity Act 2005, and the Adults with Incapacity (Scotland) Act 2000 because:

- *The research is related to the impairing condition that causes the lack of capacity or to the treatment of those with that condition*; this is critical illness caused by an underlying condition needing urgent surgery.
- *The research cannot be undertaken as effectively with people who have the capacity to consent to participate*. Patients lacking capacity due to illness severity may be a subgroup with more to gain from optimal fluid management; excluding this subgroup would limit the representativeness of the overall FLO-ELA group and reduce the generalisability of the study findings to the ultimate target clinical group.
- *The research will serve to increase knowledge of the cause, treatment or care of people with the same or similar condition and that the risks to participants will be negligible, with no significant interference with their privacy or freedom of action.* We are testing the hypothesis that goal-directed haemodynamic therapy reduces mortality after surgery, demonstrating whether this intervention is beneficial to people

with the same or similar conditions. The preceding literature suggests that the risk-benefit ratio is favourable (4). There will be no interference with privacy or freedom of action.

Having identified eligible participants at sites, research team members will assess whether the patient is capable of giving consent to trial participation.

4.1 Consent by patients

In patients with capacity, an authorised member of the team (named on the Delegation Log and with GCP training) will be responsible for obtaining written informed consent. This process will include an explanation of the aims, methods, anticipated benefits and potential hazards of the trial and provision of a Patient Information Sheet accompanied by the relevant consent form. The Principal Investigator or designee will explain to all potential participants that they are free to refuse to enter the trial or to withdraw at any time during the trial, for any reason. Patients will be given an adequate amount of time to consider their participation in the trial. Within the time available before the patient proceeds to surgery the patient will be allowed to specify the time they wish to spend deliberating, and have a second consultation if they wish to consider and discuss again. Periods shorter than 24 hours to consider the trial will be necessary due to the emergency nature of the surgery, however the person seeking consent must be satisfied that the patient has fully retained, understood and deliberated on the information given. Patients who are not entered into this trial should be recorded (including reason not entered) on the patient-screening log in the FLO-ELA Investigator Site File.

4.2 Consultation for patients lacking capacity to consent (England, Wales and Northern Ireland)

In cases where the patient lacks capacity to give informed consent and a Personal Consultee is available to advise on the presumed wishes of the patient, and there is adequate time to undergo consultation, authorised staff will explain the FLO-ELA trial and provide a Consultee Patient Information Sheet. After checking that his has been understood, if the Personal Consultee agrees that the patient would want to participate, they will be asked to sign a Consultee Declaration Form. If the Personal Consultee is not present, agreement can be obtained by telephone, and a Consultee Telephone Agreement Form will be completed. If no Personal Consultee is available, a Nominated Consultee may be approached, agreement being addressed in the same manner as for the Personal Consultee.

A Personal Consultee is defined as someone who knows the person who lacks capacity in a personal capacity who is able to advise the researcher about the person who lacks capacity's wishes and feelings in relation to the project and whether they should join the research. A Nominated Consultee is defined as someone who is appointed by the researcher to advise the researcher about the person who lacks capacity's wishes and feelings in relation to the project and whether they should join the research. This may include a member of the care team or GP, as long as they have no connection with the research project.

4.3 Emergency consent for patients lacking capacity to consent (England, Wales and Northern Ireland)

Due to the emergency nature of the surgery, and the need to proceed with medical intervention – including fluid management – there may not be a Personal or Nominated Consultee available in a timely fashion. In other cases, a Personal Consultee may be available but the urgency of the surgery means there is inadequate time for the Consultee to receive trial information and to advise on the enrolment of the person who lacks

capacity, particularly as clinical information must take priority. In these cases the authorised research team member will proceed with emergency consent using the process described in Section 32(9) of the Mental Capacity Act 2005. An independent doctor nominated by the local research team will be consulted - either in person or via telephone - and if they agree, the researcher will recruit the patient into the trial. An Emergency Consent form will be completed by the member of the research team seeking consent.

4.4 Retrospective consent (England, Wales and Northern Ireland)

If a patient subsequently recovers capacity to consent, retrospective consent will be sought. This process will use the same approach as with a first approach to patients with capacity. However, as the intervention period occurs while the patient is under anaesthesia and for only up to six hours after surgery, in almost all cases the study intervention will be completed before the patient regains capacity. In these cases consent will allow data use, but no other contact with the patient for trial interventions will be required. Patients will not be informed of their treatment group allocation until after retrospective consent is obtained. Refusal of consent at this stage should be treated as a patient withdrawal from the study, see section: **Error! Reference source not found.**. Specific Retrospective Patient Information Sheets and Retrospective Consent Forms will be used. If however a site becomes aware that the patient has a pre-existing condition which means they would never regain sufficient capacity to give informed retrospective consent, agreement of a Personal or Nominated Consultee should be sought to use the patient's data.

4.5 Recruitment of Patients lacking capacity in Scotland

The Adults with Incapacity (Scotland) Act 2000 allows recruitment of participants incapable of giving consent in a similar manner to that described under the MCA 2005 in England and Wales (see: Section 7.2.3). Where there is adequate time before surgery, consent should be obtained from any guardian or welfare attorney who has the power to consent to the patient's participation in research or, where there is no such guardian or welfare attorney, from the patient's nearest relative. Discussion of trial participation may take place in person or via telephone.

In more urgent settings with limited time available, there may not be adequate time for a guardian, welfare attorney or nearest relative to consider the trial, particularly as clinical information must take precedence. In this setting in Scotland there is currently no provision for recruitment into this type of research in the emergency settings. Therefore these patients may not be recruited to the trial.

If a patient subsequently recovers capacity to consent, retrospective consent will be sought as described above. If it becomes evident that the patient would never regain capacity, consent to use the patient's data will be sought from a guardian, welfare attorney or nearest relative.

5. Recommended intra-venous fluids

Recommended intra-venous maintenance fluids

Patients will receive one of the following fluids at 1 ml/kg/hr as maintenance fluid:

- 5% dextrose
- 4% dextrose with 0.18% NaCl (+/- KCl)
- 5% dextrose with 0.45% NaCl (+/- KCl)

It is recommended that this maintenance fluid is delivered at a fixed rate using an infusion device. Routine maintenance requirements should be satisfied in line with NICE guidance (20-30ml/kg/day of water, approximately 1mmol/kg/day of potassium, sodium and chloride and 50-100g/day of glucose) (5). In certain clinical scenarios such as electrolyte imbalance, an alternative fluid may be selected at the clinician's discretion. In obesity, the maintenance fluid rate should be adjusted to the ideal body weight, available within the NELA webtool (5).

Recommended intra-venous fluid for fluid challenges

For fluid challenges, 250ml of one of the following solutions should be used:

- "Balanced" crystalloid: Hartmann's solution (compound sodium lactate, Ringer's lactate), Plasmalyte 148.
- 0.9% sodium chloride
- Gelatin-based colloid
- Albumin

6. Cardiac Output Monitors

Investigators may only use commercially available cardiac output monitoring equipment which displays cardiac stroke volume values and accurately tracks changes in these values. Suitable technologies are:

- Arterial pressure waveform analysis (LiDCO Rapid, LiDCO Plus, Edwards Lifesciences EV1000/Hemosphere with FloTrac, Deltex ODM+/TruVue Pulse Pressure Waveform mode)
- Oesophageal Doppler (Deltex ODM/ODM+/TruVue)

7. Definitions of levels of care

The level of care should be defined according to the care the patient received rather than the location. For example, a patient receiving level 2 care in a level 3 area should be recorded as receiving level 2 care.

- Critical care level 3: includes advanced organ support e.g. invasive ventilation, renal replacement therapy.
- Critical care level 2: may include advanced cardiorespiratory monitoring (e.g. invasive arterial / central venous monitoring) and basic organ support (e.g. non-invasive ventilation, inotropic/vasoactive drug administration).
- Post-anaesthetic care unit: care within a designated area for the patients in the immediate recovery from anaesthesia. May deliver care at levels 1 to 3.
- Surgical ward (level 0/1): normal ward care without level 2 or 3 capabilities.

8. Baseline and Follow-Up Data

In hospitals in England and Wales, nearly all data described below are already collected for NELA as part of routine care, under section 251 of the NHS Act 2006. NELA data are entered on to the secure online web portal locally by the range of clinicians caring for laparotomy patients, with each specialty entering data on their area of clinical care. Existing NELA leads at each hospital monitor data completeness, addressing any

missing data and taking responsibility for completing and locking patient records. Data completeness is monitored routinely by the central NELA team and fed back to sites regularly as an audit standard. A small number of data fields will be added to the NELA web portal for FLO-ELA, only becoming activated for those patients who have given consent (or alternatives for those lacking capacity) and been randomised. Clinicians will be asked to complete these data fields prospectively, as they currently do for NELA. Research nurses will check for data completeness and accuracy of the FLO-ELA specific data fields after the intervention period. This will be monitored and actively managed throughout the trial. Not all NELA data fields are used by the FLO-ELA trial. The dataset shared is restricted to data fields that describe participant demographic and baseline characteristics, and important elements of perioperative care. Patient identifiable data are not shared from NELA to FLO-ELA; pseudonymisation (NELA and FLO-ELA trial IDs) is used for linkage.

As NELA is not commissioned in Scotland and Northern Ireland, an eCRF database will be produced with identical NELA data fields to those used in FLO-ELA. Data entry to a secure online portal will be carried out by local research teams. This database will issue an eCRF ID in the same format to the NELA ID. Local investigators in all nations will also enter identifiable data onto the secure online randomisation system, to allow linkage to national databases.

The Healthcare Quality Improvement Partnership (HQIP) are the data controller for NELA (hospitals in England and Wales) and a data sharing agreement is in place allowing sharing of pseudonymised NELA data for FLO-ELA participants as described in the participant consent materials. The FLO-ELA trial Sponsor is the data controller for the FLO-ELA data fields within the NELA database, for the data within the eCRF database used in Scotland and Northern Ireland, and for the data held within the randomisation system. FLO-ELA participant identifiable data held in the trial randomisation system will be linked to national databases to obtain outcome data including mortality and hospital (re)admission. Outcomes data will be merged with pseudonymised NELA/eCRF data to allow statistical and health economic analyses. Data sharing agreements will be established with NHS Digital and devolved nation equivalents.

8.1 Data collected during randomisation

- NHS (England), CHI (Scotland) or H&S (Northern Ireland) number*
- Date of birth*
- Gender*
- Postcode*
- NELA ID (or assigned eCRF ID in Scotland and Northern Ireland)
- Checklist to ensure the patient meets the eligibility criteria
- Patient age
- ASA score
- Indication for planned surgery
- Centre ID (collected automatically during log-in to randomisation system)

*patient identifiers are collected to allow follow up of all randomised patients.

8.2 NELA / FLO-ELA data fields

The NELA dataset is divided into seven sections that relate to the time period within the patient's perioperative pathway:

Section 1 – demographic and hospital admission data
Section 2 – preoperative (planning and care)
Section 3 – preoperative (risk stratification)
Section 4 – intraoperative care
Section 5 – surgical procedure details
Section 6 – end of surgery (includes data on FLO-ELA intra-operative intervention period. Data fields denoted 6.**F.**n)
Section 7 – postoperative (includes data on FLO-ELA post-operative intervention period) and hospital discharge. Data fields denoted 7.**F.**n)

Data fields relating exclusively to FLO-ELA participants are only visible for NELA centres participating in the FLO-ELA trial and are marked in blue in the table below. These data fields are only activated depending on the responses to fields 4.4 and 4.F.2

**1.**	**Demographics and Admission**	**Format**	**Notes**
1.1	NHS Number		**Not shared with FLO-ELA**
**1.2**	Pseudo-anonymisation		Computer generated "NELA ID"
1.3	Local patient id/hospital number		**Not shared with FLO-ELA**
1.4	Date of birth		**Not shared with FLO-ELA**
	Age on arrival		**Not shared with FLO-ELA**
1.5	Sex	▪ Male / ▪ Female	**Not shared with FLO-ELA**
1.6	Forename		**Not shared with FLO-ELA**
1.7	Surname		**Not shared with FLO-ELA**
1.8	Postcode		**Not shared with FLO-ELA**
**1.9**	Date and time the patient first arrived at the hospital/Emergency department		Admission time is 1st presentation to hospital/A&E. If the GP out of hours centre is based at the hospital A&E, then use time care was transferred from GP to the hospital. I.e. Admission time is intended to reflect the time at which the patient's care became the responsibility of the hospital.
1.10	What was the nature of this admission?	▪ Elective / ▪ Non-elective	No longer collected in NELA from 1/12/19
1.10b	If non-elective, what was the initial route of admission/assessment?	○ Assessed initially in Emergency Department<br>○ Assessed initially in "front of house" acute surgical assessment unit<br>○ Direct referral to ward by GP	No longer collected in NELA from 1/12/19
1.11	Which specialty was this patient first admitted under?	○ General surgery	No longer collected in NELA from 1/12/19

		○ General medicine<br>○ Gastroenterology<br>○ Elderly Care<br>○ Other	
1.12	Residence before this hospital admission	○ Own home/sheltered housing<br>○ Residential care<br>○ Nursing care<br>○ Unknown	No longer collected in NELA from 1/12/19
1.13a	Is this patient known to have a Learning Disability?	○	**Not shared with FLO-ELA** (introduced since start of trial recruitment) No longer collected in NELA from 1/12/2020
1.13b	Is this patient known to have an Autistic Spectrum Disorder?	○	**Not shared with FLO-ELA** (introduced since start of trial recruitment)<br>No longer collected in NELA from 1/12/2020

2	Pre-op	Format	Notes
**If the patient is returning to theatre as an emergency following previous elective surgery, all answers should relate to the emergency laparotomy, not the previous elective surgery.**			
2.1	Date and time first seen by consultant surgeon following admission with acute abdomen. If under care of a non-surgical specialty, this should be the time 1st seen after referral to general surgeons..	Date ____________(DD/MM/YYYY)<br>○ Date not known<br>Time_____________ (HH:MM)<br>○ Time not known<br>○ Not Seen	No longer collected in NELA from 1/12/19
**2.2**	Date and time that the decision was made to operate<br>*If this is unavailable please enter date and time that this patient was first booked for theatre for emergency laparotomy*	Date ____________(DD/MM/YYYY)<br>○ Date not known<br>Time_____________ (HH:MM)<br>○ Time not known	If the time is unknown for “decision made”, but date and time known for “booking”, please provide full details of the latter. If only date is known for both fields, please provide date for “decision made”.
**2.2i**	Which date and time is recorded?	○ Decision to operate<br>○ First booked for theatre	
2.3	Consultant responsible for surgical care at the time the patient was booked for surgery	(Local pick list of names with GMC number)	No longer collected in NELA from 1/12/19

	(this may be different to the operating consultant)		
**2.4**	Was there consultant surgeon input into the decision to operate?	○ Yes, consultant reviewed patient at time of decision*<br>○ Yes, following discussion with junior team member #<br>○ Decision made by junior team member **without** consultant input<br>○ Unknown	No longer collected in NELA from 1/12/19
**2.5**	NO LONGER REQUIRED	NO LONGER REQUIRED	
**2.6**	NO LONGER REQUIRED	NO LONGER REQUIRED	
**2.7**	Was an abdominal CT scan performed in the pre-operative period as part of the diagnostic work-up?	o Yes – reported by in-house consultant<br>o Yes – reported by in-house registrar<br>o Yes – reported by outsourced service<br>o Yes but NOT reported<br>o No CT performed<br>o Unknown	Combined with 2.7a from 1/12/19. Previous response options were yes/no/unknown
**2.7a**	If performed, how was this CT reported pre-operatively?	○ In-house consultant<br>○ In-house Registrar<br>○ Outsourced service<br>○ Not reported pre-operatively<br>○ Unknown	No longer collected in NELA from 1/12/19
**2.7b**	Was there a pre-operative discussion between the radiologist and the requesting team about the CT findings?	○ Yes<br>○ No<br>○ Unknown	No longer collected in NELA from 1/12/19
**2.7c**	Was there a discrepancy between the CT report and surgical findings that altered or delayed either the diagnosis or surgical management?	○ Yes<br>○ No<br>○ Unknown	No longer collected in NELA from 1/12/2020
**2.7d**	What was the Date and Time of CT Scan?	Date ____________(DD/MM/YYYY)<br>o Date not known<br>Time_____________ (HH:MM)<br>o Time not known	New question added in NELA 1/12/2020
**2.7e**	What was the Date and Time the CT Scan was reported electronically?	Date ____________(DD/MM/YYYY)<br>o Date not known<br>Time_____________ (HH:MM)<br>o Time not known	New question added in NELA 1/12/2020
**2.7f**	Was there an addendum added to	o Yes - consultant addendum to SPR report	New question added in NELA 1/12/2020

	the initial CT report which altered the patient pathway or the decision to proceed with surgery?	o Yes - in house radiologist addendum to outsourced report<br>o Yes - sub-specialist GI radiologist addendum non-GI consultant report<br>o No<br>o Unknown	
**2.8a**	Consultant Anaesthetist involvement in planning perioperative care	o Yes – seen by consultant anaesthetist in person<br>o Yes – discussion between consultant anaesthetist & other team member (of any specialty)<br>o No consultant anaesthetist input before surgery<br>o Unknown	No longer collected in NELA from 1/12/19
**2.8b**	Intensive care involvement in planning perioperative care	o Yes – seen by consultant intensivist in person<br>o Yes – discussion between consultant intensivist & other team member (of any specialty)<br>o Seen by or discussion with junior ITU team member only<br>o No intensive care input before surgery<br>o Unknown	No longer collected in NELA from 1/12/19
**2.9**	NO LONGER REQUIRED	NO LONGER REQUIRED	
**2.10**	What was the date and time of the first dose of antibiotics following presentation to hospital?	o In theatre, or<br>Date ____________(DD/MM/YYYY)<br>o Date not known<br>Time_____________ (HH:MM)<br>o Time not known<br>o Not Administered	Only relevant for non-elective admissions
**2.11a**	Was sepsis, with a NEWS2 >=5 or >=3 in any one variable or another diagnosis requiring urgent antibiotics e.g. peritonitis / perforation, suspected on admission?	o Yes<br>o No<br>o Unknown	
**2.11b**	Was sepsis, with a NEWS2 >=5 or >=3 in any one variable	o Yes<br>o No<br>o Unknown	

	and/or another diagnosis requiring urgent antibiotics e.g. peritonitis / perforation, suspected at the time the decision for surgery was made?		
**2.12**	On admission to hospital and using the Clinical Frailty Score what was the patients pre-admission frailty status assessed as being? (see help box for full pictorial explanation of each grading)	○ (1-3) - not frail<br>○ 4 - vulnerable<br>○ 5 - mildly frail<br>○ 6 - moderately frail<br>○ 7 - severely frail - completely dependent for personal care<br>○ 8 - very severely frail<br>○ 9 - Terminally ill<br>○ 0 - Not Recorded	Question wording changed from 1/12/2020 (previous version: "Using the Clinical Frailty Score (see help box), what was the patients pre-admission frailty status assessed as being?")

3	Pre-op Risk stratification	Format	Notes
**3.1**	Prior to surgery, what was the risk of death for the patient that was entered into medical record?	○ Lower (<5%)<br>○ High (>=5%)<br>○ Not documented	For information, wording of relevant standard: "An assessment of mortality risk should be made explicit to the patient and recorded clearly on the consent form and in the medical record."<br><br>If both percentage predicted mortality AND risk category are documented, please select the highest risk option<br><br>Responses changed from 1/4/19. Previous versions: lower (<5%) / high (5-10%) / highest (>10%) / Not documented
**3.1a**	If documented, how was risk assessed?	○ Objective clinical score<br>○ Clinical judgement	New combined question from 1/12/19
**3.1b**	If patient assessed to be high risk, which consultants were involved immediately preoperatively in the assessment, decision making process and care of this patient? This may be either direct or indirect care. Please mark all that apply.	○ Consultant Surgeon<br>○ Consultant Anaesthetist<br>○ Consultant Intensivist<br>○ None	New combined question from 1/12/19
**3.2**	If documented, how was this assessment of risk made? (Please select all that apply)	○ Risk prediction tool (e.g. P-POSSUM)<br>○ Clinical Judgement<br>○ Surgical APGAR<br>○ Physiological criteria<br>Other e.g. hospital policy	No longer collected in NELA from 1/12/19
**3.3**	What was the **ASA**	○ 1: No systemic disease	

	score?	○ 2: Mild systemic disease<br>○ 3: Severe systemic disease, not life-threatening<br>○ 4: Severe, life-threatening<br>○ 5: Moribund patient	
**3.4**	What was the most recent pre-operative value for serum Creatinine (micromol/l)	○ ____________<br>○ Not performed	Please enter values closest to time of booking for theatre
**3.5**	What was the most recent pre-operative value for blood lactate – may be arterial or venous (mmol/l)	○ ____________<br>○ Not performed	Please enter values closest to time of booking for theatre. Only one decimal point required.
**3.5i**	What was the highest CRP in the pre-operative period (mg/l)?	○ ____________<br>○ Not performed	No longer collected in NELA from 1/12/19
**3.5ii**	What was the lowest albumin in the pre-operative period (g/l)?	○ ____________<br>○ Not performed	
	**P-POSSUM calculation**		No longer calculated within NELA from 1/4/19
	**NELA Risk calculation**		Added to NELA from 1/4/2019
	**For questions 3.6 to 3.22 please enter values closest to time of booking for theatre in order to calculate NELA Risk score. Answers should reflect chronic *and* acute pathophysiology**.<br>**in order to calculate**		
**3.6**	Serum Sodium concentration (mmol/l)		
**3.7**	Serum Potassium concentration (mmol/l)		
**3.8**	Serum Urea concentration (mmol/l)		
**3.9**	Serum Haemoglobin concentration (g/dl)		Units must be in g/l. If results are presented as g/dl in your institution, the value should be multiplied by 10 to convert to g/l.
**3.10**	Serum White cell count (x10[illegible]9 / l)		
**3.11**	Pulse rate(bpm)		
**3.12**	Systolic blood pressure (mmHg)		
**3.13**	Glasgow coma scale		
**3.14**	Select an option that best describes this patient's **ECG**	○ No abnormalities<br>○ AF rate 60-90<br>○ AF rate >90/ any other abnormal rhythm/paced rhythm/ >5VE/min/ Q, ST or T wave abnormalities	If no investigation have been performed AND there is no clinical detail available, please select "no abnormality"
**3.15**	Select an option that best describes this	○ No failure<br>○ Diuretic, digoxin,	If CXR findings are worse than clinical findings, (or vice versa) please use worst

	patient's **cardiac signs** and chest xray appearance	antianginal or antihypertensive therapy<br>○ Peripheral oedema, warfarin Therapy or CXR: borderline cardiomegaly<br>○ Raised jugular venous pressure or CXR: cardiomegaly	score.<br>If no investigation have been performed AND there is no clinical detail available, please select "no abnormality"
**3.16**	Select an option that best describes this patient's **respiratory history** and chest xray appearance	○ No dyspnoea<br>○ Dyspnoea on exertion or CXR: mild COAD<br>○ Dyspnoea limiting exertion to < 1 Flight or CXR: moderate COAD<br>○ Dyspnoea at rest/rate > 30 at rest or CXR: fibrosis or consolidation	If CXR findings are worse than clinical findings, (or vice versa) please use worst score.<br>If no investigation have been performed AND there is no clinical detail available, please select "no abnormality"
3.16a	Patient was ventilated prior to emergency laparotomy	○ Yes<br>○ No	No longer collected within NELA
		Online web tool will automatically calculate Physiology severity score	
**3.17**	Select the **operative severity** of the intended surgical intervention (see help box for examples)	○ Major<br>○ Major+	**Major+:**<br>All colonic resections (excluding colostomy alone)<br>All gastrectomy (but not repair perforated or bleeding ulcer) Small bowel tumour resection<br>Re-operations for ongoing sepsis or bleeding<br>Laparostomy<br>Intestinal bypass<br>**Major**<br>All other procedures including: Stoma formation<br>Small bowel resection Division adhesions<br>Repair perforated or bleeding ulcer
**3.18**	Including this operation, how many operations has the patient had in the 30 day period prior to this procedure?	○ 1<br>○ 2<br>○ >2	Do not "unbundle" procedures. Examples of single procedure:<br>• Hartmann's procedure (this should not be "unbundled" as 2 procedures -sigmoid colectomy and end colostomy).<br>• Colonic resection with washout of a localised abscess would also be 1 procedure.<br>Examples of 2 procedures:<br>• Primary colonic anastomosis with a defunctioning ileostomy.<br>• Colonic resection and extensive division of adhesions.

			• Colonic resection and small bowel repair.<br><br>Example of >2 procedures:<br>Hartmann's procedure **with** resection of small bowel **with**<br>insertion of tube gastrostomy
**3.19**	Based on your clinical experience of the intended surgery, please estimate the likely ***intra*****operative blood loss** (ml)	○ <100<br>○ 101-500<br>○ 501-999<br>○ >=1000	Based on your clinical experience, please do your best to estimate the likely volume of intraoperative blood loss.
**3.20**	Please select a value that best describes the likely degree of **peritoneal soiling**	○ None<br>○ Serous fluid<br>○ Localised pus<br>○ Free bowel content, pus or blood	Based on available radiological imaging and your clinical experience, please do your best to estimate the likely degree of peritoneal soiling.
**3.21**	What severity of malignancy is anticipated to be present?	○ None<br>○ Primary only<br>○ Nodal metastases<br>○ Distant metastases	Based on available radiological imaging and your clinical experience, please do your best to estimate the extent of intra- abdominal malignancy.
**3.22**	Please select urgency of surgical intervention (see help notes for additional information, including equivalent Possum categories)	○ 3. Expedited (>18 hours)<br>○ 2B. Urgent (6-18 hours)<br>○ 2A. Urgent (2-6 hours)<br>○ 1. Immediate (<2 hours)	Based on your clinical experience this should be the maximum time that a patient could reasonably wait for surgery. These classifications are based on NCEPOD and Surviving Sepsis. The equivalent POSSUM categories are also shown.<br><br>Examples:<br>**POSSUM: Emergency (resuscitation of > 2h possible)**<br>3. Expedited (>18 hours): No SIRS or sepsis e.g. developing large bowel obstruction<br>2B. Urgent (6-18 hours): Sepsis e.g. localised abscess or obstructed hernia<br>2A. Urgent (2-6 hours): Severe sepsis e.g. intestinal perforation<br><br>**POSSUM: Emergency (immediate surgery <2h needed)**<br>1. Immediate (<2 hours): Life threatening haemorrhage and septic shock e.g. profuse GI bleed or pan-intestinal ischaemia
		Online web tool will automatically calculate Operative severity score	
3.23	Pre-op P-POSSUM predicted mortality	Calculated______________	No longer calculated within NELA from 1/4/19
3.24	Pre-op POSSUM predicted morbidity	Calculated______________	No longer calculated within NELA from 1/4/19
**3.25**	Not all NELA	○	Wording changed 1/4/19 during switch from P-

	investigations available		POSSUM to NELA risk score
**3.26**	Estimated mortality using NELA risk adjustment model	○ Calculated______________	Figure only provided if all data available

4	Intra-op	Format	Notes
**4.1**	Date and time of entry in to operating theatre/anaesthetic room (not theatre suite)	Date______________(DD/MM/YYYY)<br>Time_____________(HH:MM)<br>o Time not known	Please enter the date/time at which the patient enters the anaesthetic room OR operating theatre (for patients anaesthetisted in theatre), whichever comes first.
**4.2**	Senior surgeon grade	o Consultant<br>o Post-CCT fellow<br>o SAS grade<br>o Research Fellow / Clinical Fellow<br>o Specialty trainee / registrar<br>o Core trainee / SHO<br>o Other	This can include surgeon supervising in theatre but not necessarily scrubbed
**4.2a**	Consultant present/supervising: Name/GMC/speciality of operating or supervising consultant	(Please select consultant - Online)<br>_____________________________	If consultant not present, enter name of supervising consultant
**4.3**	Senior anaesthetist present in theatre	o Consultant<br>o Post-CCT fellow<br>o SAS grade<br>o Research Fellow / Clinical Fellow<br>o Specialty trainee / registrar<br>o Core trainee / SHO<br>o Other	
**4.3a**	Consultant present (or supervising): Name/GMC of anaesthetist	(Please select consultant - Online)<br>_____________________________	If consultant not present, enter name of supervising consultant
**4.4**	How did you provide goal directed fluid therapy?	o Patient recruited to FLO-ELA trial*<br>o Not provided<br>o Dynamic index e.g. Stroke volume, PPV, SVV<br>o Static index e.g. CVP<br>o Other, e.g. bioimpedance	PPV – pulse pressure variability<br>SVV – stroke volume variability<br>CVP – central venous pressure<br><br>*this response only available for NELA sites participating in FLO-ELA<br><br>4.4 removed from sites not participating in FLO-ELA from 1/12/19

SECTION 4 (FLO-ELA supplementary questions)		Format	Notes	Help text	Visible for CONTROL patients	Visible for INTERVENTION
**Heading**	**FLO-ELA Trial questions**					
**4.F.1**	FLO-ELA trial ID from randomisation system:	XXX-XXXXX			Y	Y
**4.F.2**	To which treatment has the patient been randomised?	CONTROL group – usual care WITHOUT cardiac output monitoring, INTERVENTION group – cardiac output-guided haemodynamic therapy as per FLO-ELA algorithm			**Y**	**Y**
**4.F.3a**	Patient weight	........kg		Measured or best estimate	Y	Y
**4.F.3b**	Patient height	........cm	RANGE: 100 - 300	Measured or best estimate	**Y**	**Y**
**4.F.3c**	Ideal body weight	Calculated		For obese patients, consider administering maintenance fluid in ml/kg based on ideal body weight.	Y	Y

5	Procedure	Format	Notes
5.1	Is this the first surgical procedure of this admission, or a complication of previous surgery within the same admission?	○ Yes- First surgical procedure after admission<br>○ No - Surgery for complication of previous elective general surgical procedure within the same admission<br>○ No – Previous 'non-abdominal/non-general	

		surgical' procedure within same admission (eg previous hip replacement)<br>○ Unknown	
**5.2**	What is the indication for surgery? (Please select all that apply)	○ Peritonitis<br>○ Perforation<br>○ Abdominal abscess<br>○ Anastomotic leak<br>○ Intestinal fistula<br>○ Phlegmon<br>○ Pneumoperitoneum<br>○ Necrosis<br>○ Sepsis<br>○ Small bowel obstruction<br>○ Large bowel obstruction<br>○ Volvulus<br>○ Internal hernia<br>○ Pseudo-obstruction<br>○ Intussusception<br>○ Incarcerated hernia<br>○ Obstructing incisional hernia<br>○ Haemorrhage<br>○ Hiatus Hernia/para-oesophageal hernia<br>○ Ischaemia<br>○ Colitis<br>○ Abdominal wound dehiscence<br>○ Abdominal compartment syndrome<br>○ Acidosis<br>○ Iatrogenic injury<br>○ Foreign body<br>○ Planned relook	
**5.3. a**	Main procedure	○ Peptic ulcer – suture or repair of perforation<br>○ Peptic ulcer – oversew of bleed<br>○ Gastrectomy: partial or total<br>○ Gastric surgery - other<br>○ Small bowel resection<br>○ Resection of Meckel's diverticulum<br>○ Colectomy: left (including sigmoid colectomy and anterior resection)<br>○ Colectomy: right (including ileocaecal resection)<br>○ Colectomy: subtotal or panproctocolectomy<br>○ Hartmann's procedure<br>○ Colorectal resection - other<br>○ Abdominal wall closure following dehiscence<br>○ Abdominal wall reconstruction<br>○ Adhesiolysis	Please note that, in accordance with NELA inclusion criteria, primary and additional procedure options vary<br><br>Please see inclusion/exclusion criteria under the "support" tab on this data collection website. They can also be downloaded from http://www.nela.org.uk/NELA_Docs
**5.3. b**	Second procedure (at same laparotomy)		
5.3. c	Third procedure (at same laparotomy)		

		○ Reduction of volvulus<br>○ Enterotomy<br>○ Stricturoplasty<br>○ Drainage of abscess/collection<br>○ Evacuation of haematoma<br>○ Debridement<br>○ Exploratory/relook laparotomy only<br>○ Haemostasis<br>○ Intestinal bypass<br>○ Laparostomy formation<br>○ Repair of intestinal perforation<br>○ Repair or revision of anastomosis<br>○ Repair of intestinal fistula<br>○ Resection of other intra-abdominal tumour(s)<br>○ Defunctioning stoma via midline laparotomy<br>○ Revision of stoma via midline laparotomy<br>○ Large incisional hernia repair with bowel resection<br>○ Large incisional hernia repair with division of adhesions<br>○ Washout only<br>○ Removal of foreign body<br>○ Not amenable to surgery<br>○ Removal of gastric band<br>○ Repair of para-oesphageal hernia<br>○ Splenectomy	
**5.3e**	Was a stoma formed (by any means)?	○ Yes<br>○ No	Added to NELA from 1/12/2019
**5.4**	Procedure approach	○ Open<br>○ Laparoscopic<br>○ Laparoscopic assisted<br>○ Laparoscopic converted to open	
**5.5**	Operative findings:<br>(Please select all that apply)<br>If unsure whether this patient is eligible for NELA please refer to help box	○ Abscess<br>○ Anastomotic leak<br>○ Perforation – peptic ulcer<br>○ Perforation – small bowel/colonic<br>○ Diverticulitis<br>○ Intestinal fistula<br>○ Adhesions<br>○ Incarcerated hernia<br>○ Volvulus<br>○ Internal hernia<br>○ Intussusception	Operative findings are intended to be best guess. There may be instances where the operative findings are such that, had these findings been known prior to surgery, the patient would not have been included in the audit. However since they have now had a laparotomy, they are still included.

		o Stricture<br>o Pseudo-obstruction<br>o Gallstone ileus<br>o Meckel's diverticulum<br>o Malignancy – localised<br>o Malignancy – disseminated<br>o Colorectal cancer<br>o Gastric cancer<br>o Haemorrhage – peptic ulcer<br>o Haemorrhage - intestinal<br>o Haemorrhage – postoperative<br>o Ulcerative colitis<br>o Other colitis<br>o Crohn's disease<br>o Abdominal compartment syndrome<br>o Intestinal ischaemia<br>o Necrotising fasciitis<br>o Foreign body<br>o Stoma complications<br>o Abdominal wound dehiscence<br>o Normal intra-abdominal findings	This is why there appear to be some findings/procedures that are under the exclusion criteria.
**5.6**	Please describe the peritoneal contamination present<br>(select all that apply)	o None or reactive serous fluid only<br>o Free gas from perforation +/- minimal contamination<br>o Pus<br>o Bile<br>o Gastro-duodenal contents<br>o Small bowel contents<br>o Faeculent fluid<br>o Faeces<br>o Blood/haematoma	
**5.7**	Please indicate if the contamination was;	o Localised to a single quadrant of the abdomen<br>o More extensive / generalised	

6	**End of Surgery**	**Format**	**Notes**
**6.1**	At the end of surgery, what risk of death was the patient documented as having?	o Lower (<5%)<br>o High (>=5%)<br>o Not documented	Responses changed from 1/4/19. Previous versions: lower (<5%) / high (5-10%) / highest (>10%) / Not documented
**6.1a**	If documented, how was risk assessed?	o Objective clinical score<br>o Clinical judgement	New combined question from 1/12/19
**6.2**	If documented, how was this assessment of risk made? (Please	oRisk prediction tool (e.g. P-POSSUM)<br>oClinical Judgement	No longer collected in NELA from 1/12/19

	select all that apply)	○Surgical APGAR<br>○Physiological criteria<br>○Other e.g. hospital policy	
**6.3**	Blood lactate – may be arterial or venous (mmol/l)	________________<br>○ Not performed	Or within 30 minutes of the end of surgery.
	**Post-operative NELA Risk calculation**<br>*Q 6.4-6.14 no longer included from Year 4 specification*		P-POSSUM changed to NELA risk score from 1/4/2019
	Physiology severity score:	(Automatically calculated)	
**6.15**	What was the operative severity? (see help box for examples)	○ Major<br>○ Major+	**Major+:**<br>All colonic resections (excluding colostomy alone)<br>All gastrectomy (but not repair perforated or bleeding ulcer)<br>Small bowel tumour resection<br>Re-operations for ongoing sepsis or bleeding Laparostomy<br>Intestinal bypass<br>**Major**<br>All other procedures including:<br>Stoma formation<br>Small bowel resection Division adhesions<br>Repair perforated or bleeding ulcer
**6.16**	Including this operation, how many operations has the patient had in the 30 day period prior to this procedure?	○ 1<br>○ 2<br>○ >2	Do not "unbundle" procedures.<br>Examples of single procedure:<br>• Hartmann's procedure (this should not be "unbundled" as 2 procedures -sigmoid colectomy and end colostomy).<br>• Colonic resection with washout of a localised abscess would also be 1 procedure.<br><br>Examples of 2 procedures:<br>• Primary colonic anastomosis with a defunctioning ileostomy.<br>• Colonic resection and extensive division of adhesions.<br>• Colonic resection and small bowel repair.<br><br>Example of >2 procedures:

			• Hartmann's procedure **with** resection of small bowel **with** insertion of tube gastrostomy
**6.17**	Please select this patient's measured intraoperative blood loss (ml)	○ <100<br>○ 101-500<br>○ 501-1000<br>○ >1000	If measured blood loss is unavailable, please estimate
6.17a	If the patient's blood loss was greater than 500mls, was Tranexamic Acid given?	○ Yes<br>○ No	**Added to NELA from 1/12/2019 Not shared with FLO-ELA**
**6.18**	Please select the option that best describes this patient's degree of peritoneal soiling	○ None<br>○ Serious fluid<br>○ Local pus<br>○ Free bowel content, pus or blood	
**6.19**	What was the level of malignancy based on surgical findings	○ None<br>○ Primary only<br>○ Nodal metastases<br>○ Distant metastases	
**6.20**	What is the NCEPOD urgency? *(see help notes for additional information, including equivalent Possum categories)*	○ 3. Expedited (>18 hours)<br>○ 2B. Urgent (6-18 hours)<br>○ 2A. Urgent (2-6 hours)<br>○ 1. Immediate (<2 hours)	Based on your clinical experience this should be the maximum time that a patient could reasonably wait for surgery. These classifications are based on NCEPOD and Surviving Sepsis. The equivalent POSSUM categories are also shown.<br><br>Examples:<br>**POSSUM: Emergency (resuscitation of > 2h possible)**<br>3. Expedited (>18 hours): No SIRS or sepsis e.g. developing large bowel obstruction<br>2B. Urgent (6-18 hours): Sepsis e.g. localised abscess or obstructed hernia<br>2A. Urgent (2-6 hours): Severe sepsis e.g. intestinal perforation<br><br>**POSSUM: Emergency (immediate surgery <2h needed)**<br>1. Immediate (<2 hours): Life threatening haemorrhage and septic shock e.g. profuse GI bleed or pan-intestinal ischaemia
	Online web tool will automatically		

	calculate Operative severity score		
6.21	Post-op P-POSSUM predicted **mortality**:	Calculated ________	No longer calculated within NELA from 1/4/2019
6.22	Post-op POSSUM predicted **morbidity**:	Calculated ________	No longer calculated within NELA from 1/4/2019
**6.23**	Not all investigations available for calculation of NELA Risk	○	P-POSSUM changed to NELA from 1/4/2019
**6.24**	Where did the patient go for continued post-operative care following surgery?	○ Ward<br>○ Critical Care (includes Level 2 HDU or Level 3 ICU)<br>○ Extended recovery area within theatres (e.g. PACU or OIR)<br>○ Enhanced care area on a normal ward<br>○ Died prior to discharge from theatre complex	"Other enhanced care area (e.g. PACU)" expanded from 1/12/2019 to include Extended recovery area within theatres (e.g. PACU or OIR) / Enhanced care area on a normal ward
**6.24a**	At the end of surgery, was the decision made to place the patient on an end of life pathway?	○ Yes<br>○ No	This is intended to identify those patients whose pathology, at the time of surgery, was such that only supportive treatment was warranted.
**6.26**	Estimated mortality using NELA risk adjustment model *(Figure only provided if all data available)*	Calculated _________	

SECTION 6	End of Surgery (FLO-ELA Trial questions)	Format	Notes	Help text	Visible for CONTROL patients	Visible for INTERVENTION patients
**Heading and subtitle**	**DURING surgery.** These questions relate to the intraoperative period. This is from the start of general anaesthesia to the end of surgery.					
6.F.1	Date and time of the start of general anaesthesia	Date____________(DD/MM/YYYY)<br>Time_____________ (HH:MM)		If the patient was fully sedated and intubated prior to arrival in the theatre suite for surgery, please indicate this. The start of the intraoperative	Y	Y

				period will be taken as the date/time when the patient arrived in the anaesthetic room/theatre (question 4.1) so please ensure this question is fully completed.		
**6.F.1a**		○ Not applicable: patient fully sedated and intubated prior to arrival in the theatre suite				
**6.F.2**	Which cardiac output monitor was used?	○ Deltex Oesophageal Doppler<br>○ Edwards EV1000/FloTrac<br>○ LiDCO Rapid<br>○ LiDCO Plus<br>○ Not used *(this is a protocol deviation)*			N	Y
**6.F.3**	Date and time of start of cardiac output-guided haemodynamic therapy:	Date_____________(DD/MM/YYYY)<br><br>Time______________ (HH:MM)			N	Y
**6.F.4**	Date and time of the end of surgery	Date_____________(DD/MM/YYYY)<br><br>Time______________ (HH:MM)			Y	Y
Subtitle:	**Maintenance fluid during surgery**				Y	Y
**6.F.5**	Fluid type	○ 5% dextrose<br>○ 4% dextrose with 0.18% NaCl (+/- KCl)<br>○ 5% dextrose with 0.45% NaCl (+/- KCl)<br>○ "Balanced" crystalloid<br>○ 0.9% sodium chloride<br>○ Other		If the type of maintenance fluid was changed during the intervention period, please select the fluid type that was given in the greatest quantity.	Y	Y
**6.F.5a**	Total maintenance fluid volume given during	......................ml	RANGE: 0 - 20000			

	surgery					
Subtitle:	**Fluid boluses during surgery**				Y	N
Subtitle:	**Cardiac output-guided fluid boluses during surgery**				N	Y
6.F.6	How many fluid boluses were given in accordance with the FLO-ELA intervention algorithm during surgery?	…….	Number format, RANGE 0-100		N	Y
**6.F.7**	Please state the volume of each of the following fluids given as boluses during surgery:				Y	Y
**6.F.7 a**	“Balanced” crystalloid	……………ml	RANGE: 0 - 20000	“Balanced” crystalloids include Hartmann’s solution (compound sodium lactate, Ringer’s lactate), Plasmalyte 147.	Y	Y
**6.F.7 b**	0.9% sodium chloride	………………..ml	RANGE: 0 – 20000		Y	Y
**6.F.7 c**	Gelatin-based colloid	………….ml	RANGE: 0 - 20000		Y	Y
**6.F.7 d**	Albumin	…………………ml	RANGE: 0 – 20000		Y	Y
**6.F.7 e**	Red blood cells	……………ml	RANGE: 0 - 20000	If exact volume is not known, please calculate from the number of units given and the average adult red cell unit volume (280ml)	Y	Y

**6.F.7 f**	Other blood products	....................ml	RANGE: 0 – 20000		Y	Y
**6.F.8**	Select any of the following drugs that were used (tick all that apply):	○ Vasopressors by bolus<br>○ Vasopressors by infusion<br>○ Inotropes by bolus<br>○ Inotropes by infusion<br>○ None of the above		For trial purposes these drugs are defined as follows:<br><br>*Vasopressors:* Metaraminol, phenylephrine, noradrenaline (norepinephrine), vasopressin<br>*Inotropes:* Ephedrine, dobutamine, dopexamine, dopamine, adrenaline (epinephrine), levosimendan	Y	Y
**6.F.9**	Was a cardiac output monitor used in a control group patient? *This is a protocol deviation*	○ No<br>○ Yes		This includes any form of cardiac output monitoring able to display stroke volume, stroke volume variation, or systolic/pulse pressure variation.	Y	N
**6.F.9 a**	**IF YES:** Date and time that cardiac output monitoring was started:	Date____________(DD/MM/YYYY)<br><br>Time_____________ (HH:MM)			Y	N
**6.F.9b**	Indication for cardiac output monitoring:	○ Patient deterioration<br>○ Other			Y	N
**6.F.9.c**	Please specify	....				

	SECTION 7: Post-op FLO-ELA trial questions.<br><br>Relating to the period DURING THE SIX HOURS AFTER surgery:				Visible for CONTROL patients	Visible for INTERVENTION patients
**7.F.1**	Which cardiac output monitor was used?	○ Deltex Oesophageal Doppler<br>○ Edwards EV1000/FloTrac<br>○ LiDCO Rapid<br>○ LiDCO Plus<br>○ Not used			N	Y
Subtitle:	**Maintenance fluid after surgery**				Y	Y
**7.F.2**	Fluid type	○ 5% dextrose<br>○ 4% dextrose with 0.18% NaCl (+/- KCl)<br>○ 5% dextrose with 0.45% NaCl (+/- KCl)<br>○ "Balanced" crystalloid<br>○ 0.9% sodium chloride<br>○ Other		If the type of maintenance fluid was changed during the intervention period, please select the fluid type that was given in the greatest quantity.	Y	Y
**7.F.2 a**	Total maintenance fluid volume given after surgery	......................ml	RANGE:<br><br>(0-20000)		Y	Y
Subtitle:	**Fluid boluses after surgery**				Y	N
Subtitle:	**Cardiac output-guided fluid boluses after surgery**				N	Y
7.F.3	How many fluid boluses were given in accordance with the FLO-ELA intervention algorithm in the six hours after surgery?	……	Number format<br><br>RANGE: 0-100		N	Y
**7.F.4**	Please state the volume of each of the following				Y	Y

	fluids given as boluses after surgery:					
**7.F.4 a**	"Balanced" crystalloid	……………ml	RANGE: 0 - 20000	"Balanced" crystalloids include Hartmann's solution (compound sodium lactate, Ringer's lactate), Plasmalyte 148.	Y	Y
**7.F.4 b**	0.9% sodium chloride	…………………ml	RANGE: 0 – 20000		Y	Y
**7.F.4 c**	Gelatin-based colloid	……………ml	RANGE: 0 - 20000		Y	Y
**7.F.4 d**	Albumin	…………………ml	RANGE: 0 – 20000		Y	Y
**7.F.4 e**	Red blood cells	……………ml	RANGE: 0 - 20000	If exact volume is not known, please calculate from the number of units given and the average adult red cell unit volume (280ml)	Y	Y
**7.F.4 f**	Other blood products	…………………ml	RANGE: 0 – 20000		Y	Y
**7.F.5**	Select any of the following drugs that were used (tick all that apply):	○ Vasopressors by bolus<br>○ Vasopressors by infusion<br>○ Inotropes by bolus<br>○ Inotropes by infusion<br>○ None of the above		For trial purposes these drugs are defined as follows:<br>*Vasopressors:*<br>Metaraminol, phenylephrine, noradrenaline (norepinephrine), vasopressin<br>*Inotropes:*	Y	Y

				Ephedrine, dobutamine , dopexamine , dopamine, adrenaline (epinephrin e), levosimend an		
**7.F.6**	Was a cardiac output monitor used in a control group patient? *This is a protocol deviation*	○ No<br>○ Yes		This includes any form of cardiac output monitoring able to display stroke volume, stroke volume variation, or systolic/pulse pressure variation.	Y	N
**7.F.6 a**	**IF YES:** Date and time that cardiac output monitoring was started:	Date____________(DD/MM/YYYY)<br><br>Time_____________ (HH:MM)			Y	N
**7.F.6 b**	Indication for cardiac output monitoring:	○ Patient deterioration<br>○ Other			Y	N
**7.F.6 c**	Please specify	....	RANGE: (max 100chars)		Y	N
**7.F.7**	Date and time of the end of cardiac output-guided haemodynamic therapy:	Date____________(DD/MM/YYYY)<br><br>Time_____________ (HH:MM)		The trial intervention should continue for six hours after surgery where possible. In cases where this has not been possible please give the reason below.	N	Y
**7.F.8a**	Was cardiac output-guided therapy stopped before six hours had elapsed after the end of	○ No<br>○ Yes			N	Y

	surgery?					
**7.F.8b**	Please state reason:	○ Patient transferred to Level 1 care<br>○ Unable to deliver ongoing intervention in Post-Anaesthetic Care Unit<br>○ Other (state):	"Other" text max 200 characters			
**7.F.9**	**For completion by the local FLO-ELA research team when patient is discharged from hospital:** Please confirm patient consent (select one choice)	○ Prospective patient consent<br>○ Prospective consultee or emergency agreement followed by retrospective patient consent<br>○ Prospective emergency agreement followed by retrospective consultee agreement (patient unable to provide consent)<br>○ Prospective consultee or emergency agreement – unable to obtain retrospective consent before discharge		Please see protocol and standard operating procedures for definitions.	Y	Y

**7**	**Post-op**	**Format**	**Notes**
**7.1**	Total length of post-operative critical care stay (rounded up to whole days)<br>*Includes both ICU and HDU stay -see help box for additional information. Do not include LOS in PACU/other enhanced recovery area*	______________<br>Number required	Each day, or part day, counts as 1 day. Hence:<br><br>a. Admitted and discharged on same day = 1 day<br>b. Admitted on Monday, discharged on Tues = 2 days<br>c. Admitted on Monday, discharged on Wed = 3 days.<br>Values should reflect actual discharge, rather than when medically fit for discharge.
7.2	NO LONGER REQUIRED		
**7.3**	For frail (CFS≥5) patients aged 65 or older, was the patient assessed by a member of the geriatrician-led multidisciplinary team during any part of the perioperative period?	○ Yes<br>○ No<br>○ Unknown	Can include physician or nurse specialist<br><br>Question wording changed from 1/12/2019 (previous version: "Was the patient assessed by a specialist from Elderly Medicine in the post-operative period?<br><br>Question wording changed

			from 1/12/2020 (previous version: "For patients aged 65 or older, was the patient assessed by a consultant geriatrician during any part of the perioperative period")
**7.4**	Within this admission, did the patient have an **unplanned or planned** return to theatre in the post-operative period following their initial emergency laparotomy?	○ Yes; unplanned return<br>○ Yes; planned return<br>○ Yes; unplanned AND planned return<br>○ No<br>○ Unknown	Question combined from 1/12/2019. Previous question only covered unplanned returns (responses yes/no)
**7.4a**	What was the main indication for the **unplanned** return to theatre	○ Anastomotic leak<br>○ Abscess<br>○ Bleeding or Haematoma<br>○ Decompression of abdominal compartment syndrome<br>○ Bowel obstruction<br>○ Abdominal wall dehiscence<br>○ Accidental damage to bowel or other organ<br>○ Stoma viability or retraction<br>○ Other<br>○ Unknown	"unplanned" added from 1/12/2019 to combine 7.4 sub-questions.
**7.4b**	NO LONGER REQUIRED	○	
**7.5**	Did the patient have an unplanned move **from the ward** to a higher level of care within 7 days of surgery? (do not include moves from HDU to ITU, or escalation from other enhanced area/PACU)	○ Yes<br>○ No<br>○ Unknown	This refers to within 7 days of their emergency laparotomy, not any prior surgery.
**7.6**	NO LONGER REQUIRED	NO LONGER REQUIRED	
**7.7**	Status at discharge	○ Dead<br>○ Alive<br>○ Still in hospital at 60 days	'Still in hospital at 60 days' option to be used when approaching an audit deadline by which all incomplete cases need to be locked
**7.8**	Date discharged from hospital	_____________<br>(DD/MM/YYYY)<br>Date required	Date of discharge, NOT date fit for discharge.<br>**Only shared with FLO-ELA for those discharged alive,** to avoid generating date of death as a direct patient identifier
**7.9**	Discharge destination	○ Own home/sheltered housing<br>○ Residential care<br>○ Nursing care<br>○ Unknown	No longer collected in NELA from 1/12/2019
	**COVID-19 Questions**		New Questions added in 2020
**7.10**	Please indicate the patient's SARS-CoV-	○ Covid positive –	

	2/COVID-19 infection status	confirmed pre-operatively<br>○ Covid positive – confirmed post-operatively<br>○ Covid negative throughout in-patient stay	
**7.11**	NO LONGER REQUIRED	NO LONGER REQUIRED	
**7.12**	NO LONGER REQUIRED	NO LONGER REQUIRED	

8.3 Outcomes data from NHS Digital or devolved nation NHS datasets

We will request hospital episode statistics and mortality data from NHS Digital for participants in England or equivalents for the devolved nations (NHS National Services Scotland Information Services Division, NSSISD and Patient Episode Database for Wales, PEDW). Prospective consent for Office of National Statistics (ONS), Hospital Episode Statistics (HES) and devolved nation equivalent data linkage will be sought before enrolment into the trial. Mortality outcomes will be derived from ONS data (for England and Wales; via NSSISD for Scotland). Duration of hospital stay and critical care stay (during the index hospital admission) will be derived from NELA data (or from a mirror electronic Case Report Form (eCRF) database in Scotland). Hospital readmissions will be derived from HES, NSSISD and PEDW data.

- Mortality at 90 days and one year after randomisation (via the ONS/NSSISD)
- Hospital admission duration including index episode and readmission to hospital as an inpatient (overnight stay) within 90 days and one year of randomisation (HES/NSSISD/PEDW)

9. Changes to statistical analysis plan

9.1 Sample size

ONS-linked NELA mortality data were available prior to trial setup for operations taking place in NELA audit year one (December 2013 – November 2014) and part of NELA audit year two (December 2014 – August 2015). Records were exported and linked to ONS in November 2015. In patients aged 50 or over, mortality within 90 days of emergency laparotomy was 19.5% in year one and 18.8% in year two. Meta-analysis of randomised trials of perioperative cardiac output-guided haemodynamic therapy suggests a relative risk reduction in mortality at longest follow-up of ~15% (RR 0.86 [95% CI 0.74 – 1.0]) (4). This would be a clinically important effect to detect, as if the intervention became routine practice for all patients undergoing emergency laparotomy it could save several hundred lives each year.

With a 5% alpha level, 90% power, and assuming a 2% dropout rate and a 19% 90-day mortality rate in the usual care arm, we initially aimed to recruit 3823 patients in each arm (7646 total) to detect a risk ratio of 0.85 (equivalent to an absolute decrease from 19% to 16.15%) for the primary outcome.

For the new primary outcome of DAOH-90, we calculated that 3138 participants (1569 per group) would be required to detect a 3.2-day increase in DAOH-90 (from mean 64.5 (SD 28.0) days in the control group to 67.7 (SD 27.1) days in the intervention group), with 90% power, a 5% alpha level, and a 2% dropout rate.

The revised sample size calculation was made without access to or knowledge of unblinded data. The parameter choices for this sample size calculation were derived from simulation as detailed in the trial Statistical Analysis Plan V4.0 and are summarised here:

Mortality

Patients who died within 90 days are assigned a DAOH-90 value of 0 (6). Prior to the revised sample size calculation based on the DAOH-90 primary outcome, pooled (control and intervention group combined) 90-day mortality was reviewed on the advice of the FLO-ELA Data Monitoring Committee in September 2019 and found to be ~12%. Therefore, the assumed control-arm event was set at ~13%, and as per the original sample size calculation, we assumed the intervention decreased mortality by 15% (relative risk 0.85) to ~11%.

Time spent in hospital

Previous studies of this intervention in higher risk (mortality >10% at longest follow-up) patient populations have found reductions in postoperative length of stay (LoS) of 1.3 days (95% CI 0.1-2.5) (4,7–13). When also considering morbidity-related hospital readmissions, a mean 2-day difference in time spent in hospital for those surviving to 90-days is realistic.

Overall effect on DAOH-90

Using statistical simulation based on the above parameters and summary hospital length of stay data from NELA (November 2020), we estimated that a 2-day difference in time in hospital for survivors and a relative risk reduction for 90-day mortality in patients in the intervention arm of 0.85 would give expected mean [SD] DAOH-90 in the Control arm of ~ 64.5 days [28.0] and in the intervention arm ~ 67.7 days [27.1], i.e. an overall 3.2-day increase in DAOH-90 in the intervention group.

This proposed effect size is realistic and of clear impact to patients and healthcare systems, representing several hundred lives saved each year, and a mean of two days less time spent in hospital for survivors if the intervention is effective and fully implemented.

9.2 Other changes to statistical analysis plan

The original version of the protocol (finalised before recruitment began) specified that subgroup analyses for age and the NELA preoperative risk score would be carried out by dichotomising the subgroup variable and modelling an interaction between the treatment variable and the dichotomised subgroup variable. We changed this so that age and NELA preoperative risk score are modelled as continuous covariates (as described in the protocol manuscript). This change was made in version 1.0 of the statistical analysis plan (SAP).

We also added a subgroup analysis by gender in version 2.0 of the SAP, analysis of the modified primary outcome and “days at home” analysis in version 3.0 of the SAP, and added analyses of the impact of COVID-19 and the estimand framework in version 4.0 of the SAP. All changes to the SAP were signed off before any contributors or members of the trial team had access to unblinded trial data.

10. Days At Home exploratory analysis

The revised primary outcome, DAOH-90, may be considered a proxy for days *at home* within 90 days (DAH-90). However, we will not have sufficiently detailed data to track each individual pathway in terms of residence outside of hospital for everyone in the database.

In order to assess if inference on DAOH-90 may be extended to DAH-90, we will analyse data for a subset of FLO-ELA participants for whom post-discharge destination ("home" or "residence other than own home") is recorded. This was recorded as part of NELA audit up to December 2019, but not thereafter. DAH-90 will be calculated in the same way as DAOH-90, except that in instances where a patient is discharged to residence other than own home, DAH-90 will be set to zero. The primary analysis on DAOH-90 will be repeated with DAH-90 for patients with available data. This will be compared against results of the primary analysis on DAOH-90 for (i) all patients (ii) subset of patients on which DAH-90 analysis carried out.

The same overarching analysis strategy will be applied with respect to all sub-group analyses and for the days-at-home analysis as that described for the primary estimand.

11. Changes to health economic analysis plan

The original version of the protocol (finalised before recruitment began) specified that the main economic analysis would be a cost-effectiveness analysis in terms of incremental cost per death avoided at 90 days post-randomisation with a secondary cost-utility analysis in terms of incremental cost per QALY gained at 90 days post randomisation. However, health policy is guided by the additional cost per QALY findings and, therefore, we will now present both analyses of incremental cost per QALY gained and per death avoided. We have also extended the economic analyses to one year post randomisation in acknowledgement of the importance of longer term differences in (quality of life-adjusted) survival for cost-effectiveness results. To support these analyses, hospital resource use data will be obtained from NHS Digital (or devolved nation equivalents) for the one year period post-randomisation. Longer-term extrapolation beyond one year follow-up post randomisation will be considered in case of remaining policy uncertainty.

12. Internal pilot phase results

The internal pilot period was taken as the first 12 months of recruitment and examined the following outcomes:

- Number of sites open and having recruited first patient
- Number of patients randomised.
- Adherence (intervention group): this is defined as a cardiac output monitor being used, and one or more cycles taken through the algorithm.
- Contamination (control group): this is defined as a cardiac output monitor being used for a patient in the control group.
- Representativeness of randomised patients compared with all eligible patients in the NELA dataset with respect to age, sex and pre-operative physiological markers.

- Control arm event rate: the Data Monitoring and Ethics Committee will assess the 90-day mortality rate in the control arm to assess whether figures used in the sample size calculation are realistic. Only patients recruited during the first five months of recruitment will be included in this analysis; this is to provide enough time to complete data linkage. The trial team will remain blinded to this event rate.

The trial feasibility phase ran from 1st September 2017 to 31st August 2018 and the results were presented to the funder on 2nd October 2018. The pre-specified stop/go criteria for progression to the remainder of the trial recruitment period and their results are summarised here:

Criterion	Pre-specified limits for progression and planned outcomes			Result and outcome
	"Green"	"Amber"	"Red"	
Number of sites open and having recruited first patient*	>90 sites: continue	70-90 sites: review site selection and initiation procedures, provide further support.	<70 sites: discuss urgently with Trial Steering Committee and funder, considering all options including discontinuation.	40 sites active. TSC and funder recommended recovery plan to increase site and participant numbers
Participants randomised*	>80% of target (1426 participants): continue	50-80% of target (890 – 1426 participants): consider recruitment strategies (opening more centres, further training and support).	<50% of target (<890 participants): discuss urgently with TSC and funder, considering all options including discontinuation.	544 participants randomised. Noted that the per-site recruitment rate was very close to target therefore concluded to be predominantly due to inadequate active sites. Addressed with recovery plan as above.
Adherence (intervention group)	>90%: continue	80-90%: consider options such as re-training staff, providing further support, closing problem sites.	<80%: discuss urgently with TSC and funder, considering all options including discontinuation.	91.6% adherence rate: continue
Contamination (control group)	<10%: continue	10-20%: consider options such as re-training staff, providing further support. Individual sites with contamination	>20%: discuss urgently with TSC and funder, considering all options including discontinuation.	5.3% contamination rate: continue

		rates over 10% may be closed at the end of the pilot period.		
Representativeness of recruited participants	Small differences in all variables (<5 years difference in age, <10% difference in gender, <10% difference in pre-operative mortality risk score): continue.	Large difference in one or more variables: consider strategies to target specific groups.		Data available for 513 participants recruited to FLO-ELA, compared with 4449 patients within NELA at recruiting sites. In FLO-ELA vs. NELA: mean age 70.8yrs vs. 70.4yrs (difference [95% confidence interval] = 0.5 [-0.4, 1.4]), % females 54 vs. 52.9 (difference 1.1 [-3.2, 5.5]), NELA pre-operative mortality risk score mean 9.0 vs. 11.7 (difference -2.7 [-3.7, -1.6]). Outcome: continue

*Based on original targets of 100 recruiting sites and 7646 target sample size prior to Covid-19-related pause in recruitment.

The control arm event rate (mortality at 90-days after randomisation) was reviewed by the DMEC. The rate was not revealed to the TSC, trial team or funder to avoid unblinding. The DMEC issued a statement that the control group event rate was consistent with pre-trial assumptions and did not recommend any changes to the planned sample size at this stage.

13. Supplementary File References

1. National Confidential Enquiry into Patient Outcome and Death. NCEPOD Revised Classification of Operation [Internet]. 2004. Available from: http://www.ncepod.org.uk/pdf/NCEPODClassification.pdf

2. Pinkney TD, Calvert M, Bartlett DC, Gheorghe A, Redman V, Dowswell G, et al. Impact of wound edge protection devices on surgical site infection after laparotomy: multicentre randomised controlled trial (ROSSINI Trial). BMJ. 2013;347:1–13.

3. Kay R, Siriwardena A. The process of informed consent for urgent abdominal surgery. J Med Ethics. 2001 Jun;27(3):157–61.

4. Pearse RM, Harrison DA, MacDonald N, Gillies MA, Blunt M, Ackland G, et al. Effect of a perioperative, cardiac output-guided hemodynamic therapy algorithm on outcomes following

major gastrointestinal surgery: a randomized clinical trial and systematic review. JAMA. 2014 May 19;311(21):2181–90.

5. NICE. Intravenous fluid therapy in adults in hospital (Clinical Guidance 174) [Internet]. 2014 [cited 2014 Apr 16]. Available from: http://www.nice.org.uk/
6. Jerath A, Austin PC, Wijeysundera DN. Days Alive and Out of HospitalValidation of a Patient-centered Outcome for Perioperative Medicine. Anesthesiology. 2019 Jul 1;131(1):84–93.
7. Boyd O, Grounds RM, Bennett ED. A randomized clinical trial of the effect of deliberate perioperative increase of oxygen delivery on mortality in high-risk surgical patients. JAMA. 1993 Dec 8;270(22):2699–707.
8. Calvo-Vecino JM, Ripollés-Melchor J, Mythen MG, Casans-Francés R, Balik A, Artacho JP, et al. Effect of goal-directed haemodynamic therapy on postoperative complications in low–moderate risk surgical patients: a multicentre randomised controlled trial (FEDORA trial). British Journal of Anaesthesia [Internet]. 2018 Feb [cited 2018 Mar 11]; Available from: http://linkinghub.elsevier.com/retrieve/pii/S0007091217542075
9. Lobo SM, Salgado PF, Castillo VG, Borim AA, Polachini CA, Palchetti JC, et al. Effects of maximizing oxygen delivery on morbidity and mortality in high-risk surgical patients. Crit Care Med. 2000 Oct;28(10):3396–404.
10. Pearse R, Dawson D, Fawcett J, Rhodes A, Grounds RM, Bennett ED. Early goal-directed therapy after major surgery reduces complications and duration of hospital stay. A randomised, controlled trial [ISRCTN38797445]. Critical Care. 2005;9(6):R687–93.
11. Sandham JD, Hull RD, Brant RF, Knox L, Pineo GF, Doig CJ, et al. A randomized, controlled trial of the use of pulmonary-artery catheters in high-risk surgical patients. N Engl J Med. 2003 Jan 2;348(1):5–14.
12. Shoemaker WC, Appel PL, Kram HB, Waxman K, Lee TS. Prospective trial of supranormal values of survivors as therapeutic goals in high-risk surgical patients. Chest. 1988 Dec;94(6):1176–86.
13. Wilson J, Woods I, Fawcett J, Whall R, Dibb W, Morris C, et al. Reducing the risk of major elective surgery: randomised controlled trial of preoperative optimisation of oxygen delivery. BMJ. 1999 Apr 24;318(7191):1099–103.
